# Trends, Projections, and Regional Disparities of Maternal Mortality in Africa (1990–2030): An ARIMA Forecasting Approach

**DOI:** 10.3390/epidemiologia4030032

**Published:** 2023-08-29

**Authors:** Luc Onambele, Sara Guillen-Aguinaga, Laura Guillen-Aguinaga, Wilfrido Ortega-Leon, Rocio Montejo, Rosa Alas-Brun, Enrique Aguinaga-Ontoso, Ines Aguinaga-Ontoso, Francisco Guillen-Grima

**Affiliations:** 1 School of Health Sciences, Catholic University of Central Africa, Yaoundé 1110, Cameroon; onambele.luc@ess-ucac.org; 2 Department of Health Sciences, Public University of Navarra, 31008 Pamplona, Spain; saraguillen.sg@gmail.com (S.G.-A.); lguillen@alumni.unav.es (L.G.-A.); rosamaria.alas@unavarra.es (R.A.-B.); 3 Department of Nursing, Suldal Sykehjem, 4230 Sands, Norway; 4 Department of Surgery, Medical and Social Sciences, University of Alcala de Henares, 28871 Alcalá de Henares, Spain; wilfrido.ortega@edu.uah.es; 5 Department of Obstetrics and Gynecology, Institute of Clinical Sciences, University of Gothenburg, 413 46 Gothenburg, Sweden; rocio.montejo.rodriguez@gu.se; 6 Department of Obstetrics and Gynecology, Sahlgrenska University Hospital, 413 46 Gothenburg, Sweden; 7 Department of Sociosanitary Sciences, University of Murcia, 30120 Murcia, Spain; aguinaga@um.es; 8 Area of Epidemiology and Public Health, Healthcare Research Institute of Navarre (IdiSNA), 31008 Pamplona, Spain; 9 CIBER in Epidemiology and Public Health (CIBERESP), Institute of Health Carlos III, 46980 Madrid, Spain; 10 Department of Preventive Medicine, Clínica Universidad de Navarra, 31008 Pamplona, Spain

**Keywords:** Africa, ARIMA, machine learning, maternal mortality rate, joinpoint regression analysis, mortality, trends, forecasting

## Abstract

With the United Nations Sustainable Development Goals (SDG) (2015–2030) focused on the reduction in maternal mortality, monitoring and forecasting maternal mortality rates (MMRs) in regions like Africa is crucial for health strategy planning by policymakers, international organizations, and NGOs. We collected maternal mortality rates per 100,000 births from the World Bank database between 1990 and 2015. Joinpoint regression was applied to assess trends, and the autoregressive integrated moving average (ARIMA) model was used on 1990–2015 data to forecast the MMRs for the next 15 years. We also used the Holt method and the machine-learning Prophet Forecasting Model. The study found a decline in MMRs in Africa with an average annual percentage change (APC) of −2.6% (95% CI −2.7; −2.5). North Africa reported the lowest MMR, while East Africa experienced the sharpest decline. The region-specific ARIMA models predict that the maternal mortality rate (MMR) in 2030 will vary across regions, ranging from 161 deaths per 100,000 births in North Africa to 302 deaths per 100,000 births in Central Africa, averaging 182 per 100,000 births for the continent. Despite the observed decreasing trend in maternal mortality rate (MMR), the MMR in Africa remains relatively high. The results indicate that MMR in Africa will continue to decrease by 2030. However, no region of Africa will likely reach the SDG target.

## 1. Introduction

The right to give birth safely is a fundamental human right [1,2]. Maternal mortality is a severe global health issue in Africa [1]. According to United Nations (UN) data, around 287,000 women globally lose their lives annually due to maternal complications, amounting to roughly one death every two minutes [3]. As defined by the World Health Organization (WHO), maternal death refers to the death of a woman during pregnancy or within 42 days of pregnancy completion due to complications or circumstances directly associated with the pregnancy [4]. Maternal mortality is increasing in developed countries like the United States, especially among minorities [5,6]. Despite interventions, preventable maternal deaths in Africa remain high, majorly caused by complications such as hemorrhage, eclampsia, sepsis, and delivery complications. HIV/AIDS often exacerbates these conditions [7,8]. The main causes of maternal mortality in Africa are eclampsia and hemorrhage [9]. Institutional delivery is vital, as childbirth facilitated by trained healthcare professionals can significantly reduce maternal mortality. However, systemic issues like staffing shortages, accessibility, and service quality seriously challenge many African regions [10,11]. This study also explores the broader socioeconomic context of Africa by examining the effects of global events, such as the economic downturn of 2007, on maternal health outcomes [12,13].

The Millennium Development Goals (MDGs) aimed to achieve a 75% reduction in the maternal mortality rate (MMR) between 1990 and 2015 [14,15,16,17]. However, at a meeting in Luanda, Angola 2014, the Health Ministers of Africa acknowledged that only four African countries—Cape Verde, Equatorial Guinea, Eritrea, and Rwanda—had achieved this target [18]. The United Nations Sustainable Development Goals aim to reduce the global maternal mortality ratio to less than 70 per 100,000 live births by 2030 [19]. Achieving this goal requires strategic investments in healthcare infrastructure, educational enhancement, and significant policy changes [20]. These interventions should primarily focus on improving the accessibility and quality of maternal health services. Creating maternal health services accessible to the population is essential to reduce maternal mortality. As per the guidelines of the UN regarding emergency obstetric and neonatal care, there should be a minimum of five facilities providing such care for every 500,000 people. Out of these, at least one facility must be a comprehensive emergency obstetric and neonatal facility [21]. Researchers and experts have utilized these indicators to monitor the progress of maternal health services in Africa [22,23] and other continents [24,25,26].

However, past research leaves much to be desired. Previous studies have several limitations that this study aims to address. For instance, data quality and completeness have been issues in several previous studies, primarily due to the limited resources for comprehensive and regular data collection and validation in many parts of Africa [27,28]. This data deficiency leads to potential biases and uncertainties in the estimated MMRs and trends [28,29,30].

Additionally, there is a dearth of research examining the variations in MMR across the various African regions and their underlying causes [28,30]. The diversity of healthcare systems, socioeconomic contexts, and policy environments across Africa is considerable [30], yet many studies have treated Africa as a homogenous entity. This may lead to conclusions and recommendations that are not sufficiently tailored to the unique circumstances of each region.

Furthermore, most studies have focused more on the impact of direct health interventions, often neglecting the broader socioeconomic factors that play a crucial role in maternal health outcomes. Factors such as literacy rates, income level, women’s status, and other social determinants of health are under-studied.

Lastly, the temporal perspective has been under-represented in the literature, with a lack of robust forecasting of future trends and the impact of historical and future global events. This limits the utility of research findings for strategic planning and policymaking, as it does not provide a forward-looking view.

By acknowledging these limitations, this study seeks to provide a more holistic understanding of maternal mortality in Africa, considering regional variations, socioeconomic determinants, and future trends. In this way, our findings will contribute to a more nuanced understanding that will facilitate the development of more effective, targeted interventions.

There is also a critical need to enhance women’s access to the continuum of care, particularly post-natal care, and advocate for village-based healthcare systems to mitigate geographical barriers, thereby improving neonatal outcomes in remote areas [31]. The present study uses data from the World Bank’s public database to study the MMR trends across Africa and forecast future patterns using the ARIMA and Holt models [32,33] Forecasting the MMR is crucial for policymakers, international organizations, and NGOs in planning health strategies [34]. The findings of our study will underline the urgent need for a holistic approach to improving maternal health in Africa, emphasizing data analysis, institutional delivery, and the effects of socioeconomic factors. The goal is to inspire additional research and policy discussions to help Africa progress toward achieving sustainable development goals in maternal health.

The ARIMA model has demonstrated significant utility in epidemiological studies due to its adeptness in managing complex datasets. This has been exemplified in several contexts: it has been used to forecast measles immunization coverage, providing policymakers with valuable information to allocate resources effectively [35]. It was instrumental in simulating and forecasting the influenza epidemic process in Ukraine [36], thereby contributing to the timely development of anti-epidemic measures, and it successfully predicted the potential impact of COVID-19 vaccination on reducing maternal deaths in Mexico [37]. In this study, we leverage the ARIMA model, following these precedents, to analyze trends, projections, and regional disparities of maternal mortality in Africa from 1990 to 2030.

In response to the identified gaps in the existing literature and the pressing need to further our understanding of maternal mortality in Africa, this study is guided by two central research questions, considering the complex realities that characterize maternal health in Africa: Firstly, we seek to comprehensively analyze the trends, disparities, and future projections of maternal mortality rates across various African regions, considering the diverse healthcare systems, socioeconomic contexts, and policy environments. This includes examining the underlying causes and factors contributing to these trends and disparities. Our first research question is what are the trends, disparities, and future projections of maternal mortality rates across various African regions? Secondly, we aim to investigate how broader socioeconomic contexts and significant global events, such as the 2007 economic downturn, have historically affected and will likely continue to influence these trends and projections. So, our second research question is how do socioeconomic context and global events, like the 2007 economic downturn, affect these trends and projections? These research questions are instrumental in shaping this study’s methodology and analyses, allowing us to present a nuanced, region-specific understanding that can inform targeted interventions and policy decisions.

Our hypotheses, informed by our understanding of the persistently high maternal mortality rates in Africa, despite global efforts and the impact of broader socioeconomic factors on health outcomes, are as follows: While there has been a decrease in maternal mortality rates in Africa, disparities across the regions persist and, without significant interventions, will continue to do so till 2030. Socioeconomic contexts and global events significantly influence maternal mortality rates, affecting the pace and nature of progress in different regions. These questions and hypotheses guide our exploration of the historical trends of maternal mortality rates from 1990 to 2015 and the projected trends up to 2030. By conducting this analysis, we aim to provide a comprehensive picture of maternal mortality in Africa, supporting the creation of targeted interventions and guiding policy discussions. The goal is to inspire additional research and policy discussions to help Africa progress toward achieving sustainable development goals in maternal health.

## 2. Materials and Methods

MMRs and population data were extracted from all African countries’ World Bank mortality databases from 1990 to 2015 [19,38]. The World Bank database was elaborated by the Maternal Mortality Estimation Inter-Agency Group (MMEIG), which utilized various data sources to provide maternal mortality rates. Primary sources were country-specific data comprising absolute numbers of maternal deaths, maternal deaths per 100,000 live births, and the proportion of deaths among women of reproductive age due to maternal causes. When available, these metrics were typically extracted from Civil Registration and Vital Statistics (CRVS), which were preferred for their comprehensiveness and representativeness. Additionally, specialized studies conducted by countries to ascertain the potential underreporting of maternal deaths in their CRVS systems contributed to the data. Without reliable CRVS systems, alternate data sources such as population-based surveys like the Demographic and Health Surveys Multiple Indicator Cluster Surveys and miscellaneous studies were employed. The World Bank also incorporated covariates into the estimation model, such as GDP, the general fertility rate (GFR), and the coverage of skilled attendants at birth (SAB) to guide the projection of trends in sparse-data settings. Sources for these covariates included the World Bank itself for GDP data, the United Nations Population Division (UNPD) for GFR, and UNICEF for SAB data. Based on the available data and inherent uncertainties, adjustments were made to the collected data to account for systematic and random errors, thereby improving the precision of the final maternal mortality rates [39].

Thus, we extracted the data from the database up to 2015, making it the latest year included in our analysis. The original structure of the dataset placed each African country in a row, with the columns representing the yearly data for each country. In this configuration, the first column corresponded to the country names, and the subsequent columns contained the annual maternal mortality rates starting from the year 1990 through to 2005. It was necessary to transpose the data. Transposing is a method commonly used in data manipulation where the rows and columns are interchanged; the rows become columns and vice versa. This process reorganized our data so that each row represented a year while each column corresponded to an African country. The data transposition was performed using Microsoft Excel. Once transposed, each row of our dataset corresponded to a year (from 1990 to 2005), while the columns represented the maternal mortality rates for each African country.

**Figure 1:**
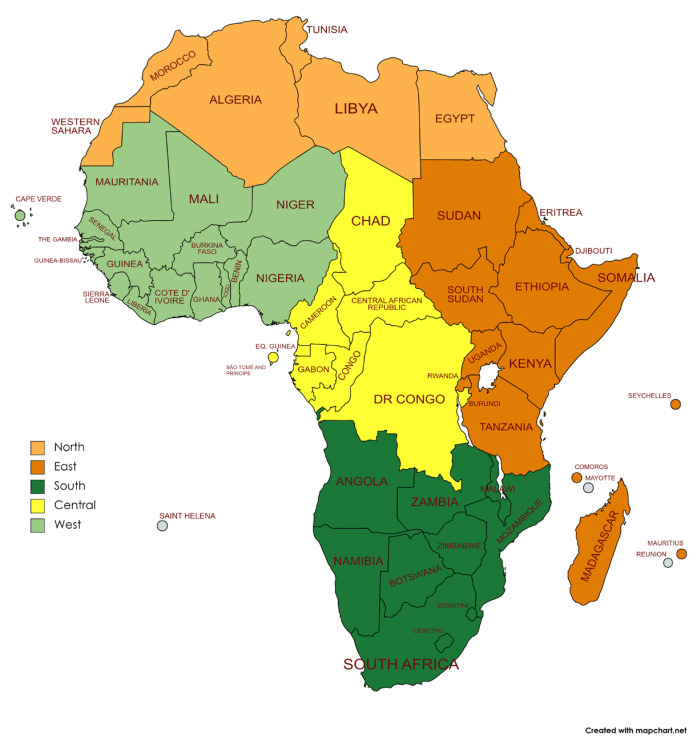
Regions of the African Union, according to the African Union classification.

We used the five regions of the African Union classification (Figure 1) [40,41]. We excluded British and French territories from our analysis as they were not part of the African Union. We excluded Seychelles and Western Sahara from the analysis due to the lack of data.

In this study, when ‘Africa’ is used alone, it refers to the entire African continent, including all associated islands, notably Madagascar and Cape Verde. However, when accompanied by directional terms such as ‘North’, ‘South’, ‘East’, ‘Central’, and ‘West’, the term refers to the specific regions within the continent.

The annual mortality rates for each region and Africa were estimated by weighting the region of each country’s MMR with its population [38] The time series were evaluated for autocorrelation using the Durbin–Watson test. We used this test to evaluate two contrasting hypotheses: H0 (null hypothesis), stating that the residuals are not autocorrelated, and HA (alternative hypothesis), which suggests the existence of autocorrelation within the residuals.

### 2.1. Joinpoint Regression

We performed a joinpoint regression to detect changes in the trends. We estimated the annual percentage change (APC) to describe the magnitude of the change in each trend and calculated the 95% confidence intervals (95% CI).

### 2.2. Arima Models

We used autoregressive integrated moving average (ARIMA) models to predict MMR. MMR was the working variable to be forecasted. We computed separate models for each region. The ARIMA model involves three parameters, *p*, *d*, and *q*, where *p* is the model’s autoregressive (AR) part. *P* integrates the effect of lag value; *d* represents the number of differences needed for making the model stationary, and *q* is the moving average (MA) of the number of lagged forecast errors in the prediction equation [42,43]. We used autocorrelation (ACF) and partial autocorrelation (PACF) graphs to check autocorrelation. We used the Bayesian Information Criterion (BIC) to select the best model. Finally, we applied the McLeod–Li test to check the stationarity of the model residuals. We first checked the stationarity of the data from 1990 onwards. After confirming stationarity, we used the autocorrelation function (ACF) and partial autocorrelation function (PACF) plots to determine appropriate values for the autoregressive (*p*) and moving average (*q*) components of the ARIMA model. We then fit the ARIMA model to the data and checked the residuals to ensure they were white noise.

The autoregressive integrated moving average (ARIMA) model is a generalization of the autoregressive moving average (ARMA) model and is designed to model time series data. ARIMA models take into account three aspects: autoregression (AR), differencing (I), and moving average (MA). In this study, different ARIMA models were selected for different regions based on the specific characteristics of the data from those regions.

The AR aspect models the dependency between observation and several lagged observations. The ‘I’ aspect refers to the number of difference operations needed to make the time series stationary, i.e., data values are not a function of time. The ‘MA’ aspect models the dependency between an observation and a residual error from a moving average model applied to lagged observations. ARIMA models are beneficial for forecasting when the data show evidence of non-stationarity, where the mean, variance, or covariance vary over time. Non-stationarity is particularly relevant for this study due to the nature of maternal mortality rates, which are likely to change over time due to improvements in healthcare, policy changes, or other factors.

The ARIMA model’s parameters (*p, d, q*) were determined for each region based on the behavior of the data. The p parameter is determined by the partial autocorrelation plot (PACF), while the q parameter is determined by the autocorrelation function (ACF). The parameter d, which refers to the differencing required to achieve stationarity, is determined based on the nature of the data. The research also adapted the ARIMA model to accommodate regional disparities, thus tailoring the ARIMA parameters to suit the characteristics of the data from each region. This adaptation allowed for a more accurate projection of maternal mortality rates and helped capture the variation across different regions. The Bayesian Information Criterion (BIC) was used to assess the quality of the model fit. The BIC is a statistical measure used to compare different models, with a lower BIC indicating a better model fit. The stationary R-squared value, which measures the proportion of variance in the dependent variable that can be predicted from the independent variable, was also used to evaluate the performance of the ARIMA models. For the region of South Africa, the study used data from 2003 onwards to fit the ARIMA model due to a notable structural change in the data around 2003, indicating a shift in the dynamics of the MMR. This highlights the flexibility of the ARIMA model, which can be adapted to capture changing trends in data. The study also compared the ARIMA models to forecasts produced using the Holt Exponential Smoothing method and machine learning.

### 2.3. Holt Exponential Smoothing

We also used the Holt Exponential Smoothing method to forecast African maternal mortality trends [44]. This method offers an approach for time series forecasting that considers both the level and the trend, making it particularly suitable for trends with non-stationary data. The model’s appropriateness was assessed by scrutinizing the diagnostic results of residuals and determining the mean absolute percentage error (MAPE) used to measure the accuracy of these forecasts. The final model was then used to forecast the future maternal mortality trends in Africa. We computed 95% confidence intervals. Due to the nature of mortality rates, which cannot be negative, we adjusted the confidence interval by setting the lower bound to zero if it fell below that value.

### 2.4. Machine Learning Forecasting

Our research employed a machine learning system, specifically the Prophet Forecasting Model (PFM) developed by Facebook [45]. Prophet decomposes the time series data into various components and learns their underlying relationships and dependencies based on their evolution in historical data. This understanding projects future alterations in these components, leading to a trend forecast. The model inherently incorporates uncertainty, providing an uncertainty interval with each forecasted point. We used Prophet to analyze historical data and forecast maternal mortality rates in Africa from 1990 to 2015, extending predictions to 2030.

Instead of segregating the data into a training set and a test set, Prophet utilizes all the available data for modeling and projects future dates beyond the existing data range. We applied a cross-validation approach to verify the forecast’s accuracy and reliability. In this method, we designated an initial time series period as a training set and assigned a holdout set for validation. The model was thus trained on data from 1990 to 2009, and predictions were subsequently generated from 2010 to 2015. These predictions were compared to actual values to compute prediction error, utilizing a walk-forward validation method prevalent in time-series forecasting. We then calculated the mean absolute percentage error (MAPE). In the analysis of the Prophet Model, uncertainty intervals (UI) were used instead of the traditional confidence intervals (CIs). UIs were computed to capture the range within which the true value of the MMR is expected to lie, considering both aleatory uncertainty (associated with inherent randomness) and epistemic uncertainty (resulting from lack of knowledge). This method provides a broader understanding of the potential variability and uncertainty in the projections. UIs were estimated at the 95% level, meaning that the true value is expected to fall within the specified range 95% of the time. The utilization of UIs in this context offers a more comprehensive picture of the uncertainty inherent in forecasting future MMR, aligning with the complexity and multifaceted nature of the phenomenon being modeled.

### 2.5. Software

We performed the computations using IBM SPSS v17 and Joinpoint Regression Program, Version 4.9.0.1, February 2022, from the Statistical Research and Applications Branch, National Cancer Institute of the United States. The Holt forecasting was performed in R (version 4.1.0) using the ‘forecast’ package (version 8.21) [46,47]. We utilized the ‘Prophet’ package (version 1.0) in RStudio (version 2023.06.1+524) to compute the PFM [45].

## 3. Results

The data from international standardized sources may differ from some values collected at the national level via the routine data collection system [27]. Nevertheless, we used these data because they were derived from standard and validated methodologies [48].

**Figure 2:**
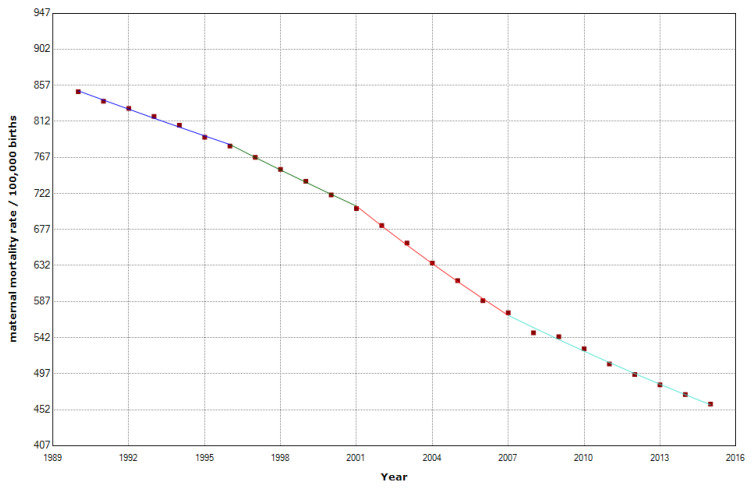
Maternal mortality trends in Africa (1990–2015) indicating joinpoints at the transitions between colored lines.

The MMR declined from 788 deaths per 100,000 births in 1990 to 392 deaths per 100,000 births in 2015 (Figure 2). However, 205,670 women still died in Africa in 2015. Most maternal deaths (203,000) occurred in Sub-Saharan Africa [19] We have identified three join points in 1996, 2001, and 2007, indicating the presence of four distinct phases characterized by significant shifts in the maternal mortality trend.

Table 1 shows that there was a significant decline in MMR by a significant APC (−2.6%) (*p* < 0.001). In the first period, 1990–1996, there was a slight decrease, with an APC of −1.4%. A series of stages followed in which the APC reduction in maternal mortality rates was progressively higher and higher. The accelerated trend was interrupted in 2007 when there was a slowdown in the APC. The APC changed from −3.5% in 2001–2007 to −2.1% in 2007–2015.

Table 2 presents the parameters of the ARIMA models, the stationary R-squared, and the ARIMA models’ normalized Bayesian Information Criterion (BIC).

**Figure 3:**
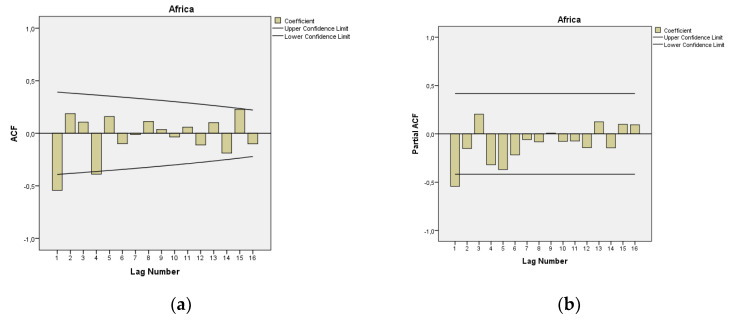
Maternal mortality in Africa (**a**) autocorrelation (ACF) (**b**) and partial autocorrelation (PACF).

In Figure 3, we present the ACF and PACF of the temporal series of MMR in Africa. The ARIMA model of MMR in Africa was ARIMA (1,1,1). In the ARIMA model, the value of the stationary R-squared was 0.277, and the normalized Bayesian Information Criterion (BIC) was 3.662.

**Figure 4:**
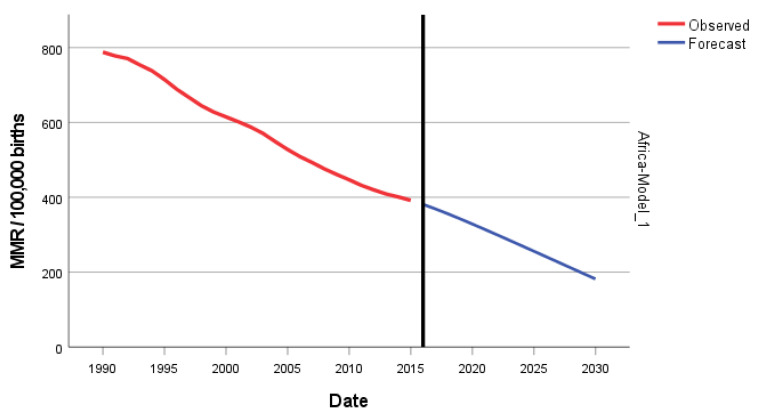
Evolution and forecasting of MMR in Africa.

In Figure 4, we present the forecasting of MMR for Africa until 2030. The predicted forecast of MMR in Africa for 2030 is 182 deaths per 100,000 births.

**Figure 5:**
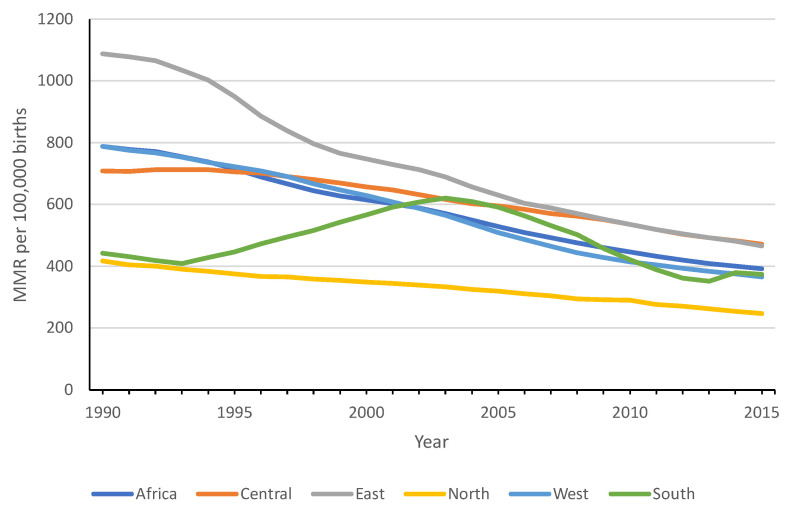
Evolution of regional maternal mortality rates (1990–2015).

According to Figure 5, there has been a noticeable change in the regional maternal mortality rate (MMR) over time. The gap between the regions with the highest and lowest MMR experienced a decline from 670 per 100,000 births in 1990 to 224 in 2015, indicating a reduction in interregional disparities as time progressed.

The North and South Africa regions consistently remain with lower MMRs throughout the period. There has been a convergence between Central and West Africa. Finally, the East African region, which started with extremely high levels, has experienced a sharp decline. Its rates tend to converge with those of the Southern Africa region.

### 3.1. North Africa

**Figure 6:**
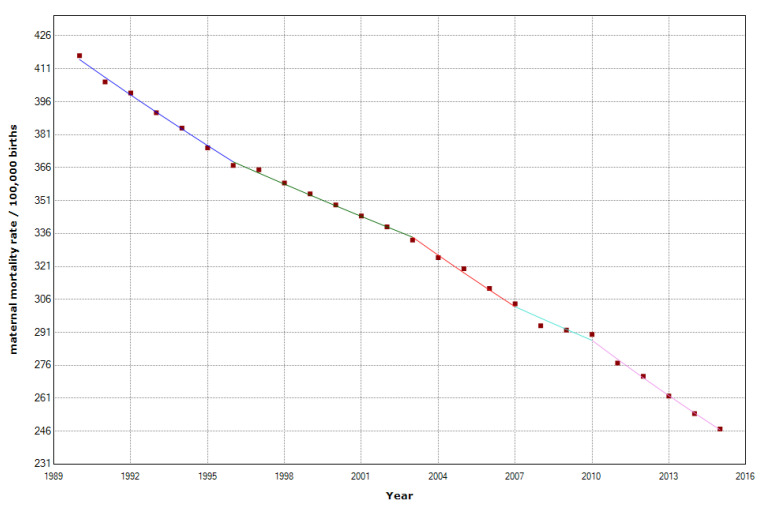
Maternal mortality trends in North Africa (1990–2015) indicating joinpoints at the transitions between colored lines.

The overall MMR decreased from 417 to 247 per 100,000 births in North Africa, which is a decrease of 40.8%. We found a statistically significant decrease in the MMR, with an APC of −1.9% (95% CI −2.0; −1.8). We detected four joinpoints in 1996, 2003, 2007, and 2010 (Table 3, Figure 6).

**Figure 7:**
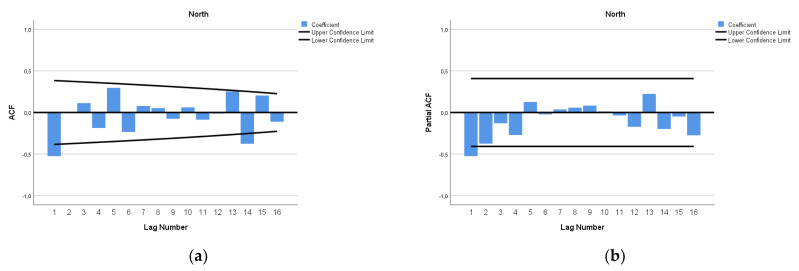
Maternal mortality in North Africa (**a**) autocorrelation (ACF) (**b**) and partial autocorrelation (PACF).

**Figure 8:**
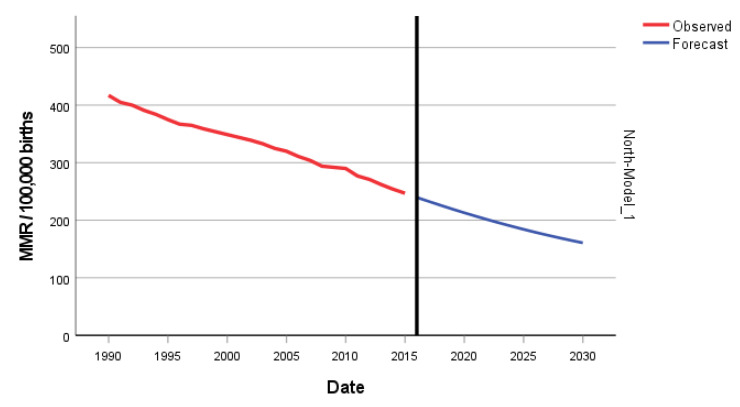
Evolution and forecasting of MMR in North Africa.

The ARIMA model of MMR in North Africa was (1,2,0). In Figure 7, we present the ACF and PACF of the temporal series of MMR in North Africa. In the ARIMA model, the value of the stationary R-squared was 0.289, an R-squared of 0.994, and the normalized Bayesian Information Criterion (BIC) was 3.037 (Table 2). The predicted forecast of MMR in North Africa in 2030 is 161 deaths per 100,000 births (Figure 8).

### 3.2. East Africa

**Figure 9:**
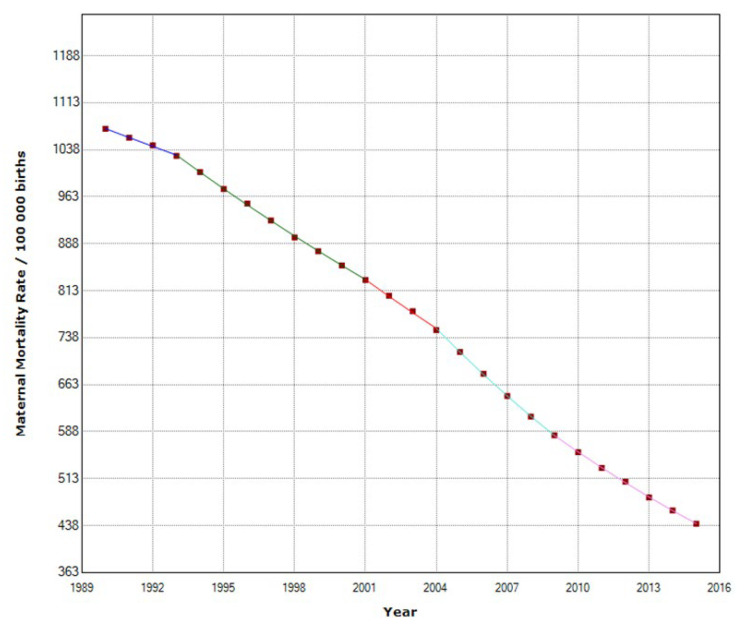
Maternal mortality trends in East Africa (1990–2015) indicating joinpoints at the transitions between colored lines.

In East Africa, MMR decreased from 1087 to 466 maternal deaths per 100,000 births (Table 3). East Africa was the region with the highest decrease during the study period. MMR was reduced by 57.1%. Maternal mortality decreased annually by 3.6%, with four join points in 1993, 2001, 2004, and 2009 (Figure 9).

**Figure 10:**
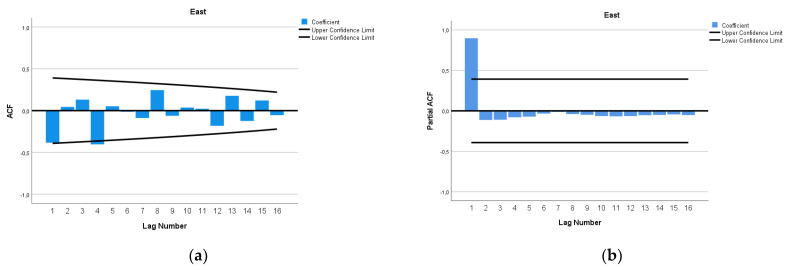
Maternal mortality in East Africa (**a**) autocorrelation (ACF) (**b**) and partial autocorrelation (PACF).

The ARIMA model of MMR in East Africa was (1,3,0). In Figure 10, we present the ACF and PACF of the temporal series of MMR in North Africa. In the ARIMA model, the value of the stationary R-squared was 0.148, an R-squared of 0.997, and the normalized Bayesian Information Criterion (BIC) was 4.696.

**Figure 11:**
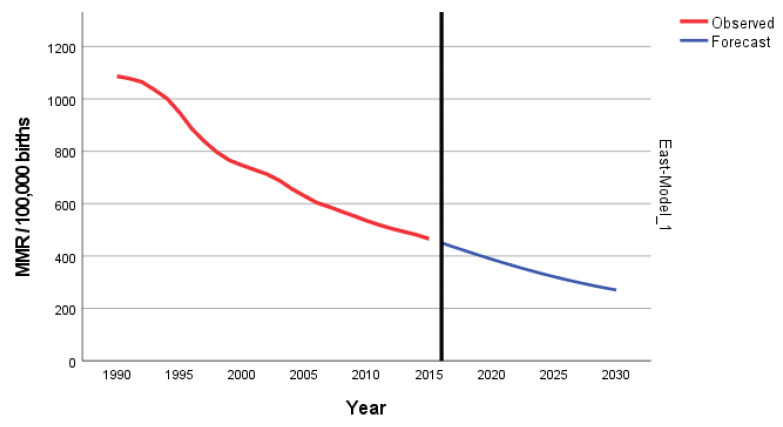
Evolution and forecasting of MMR in East Africa.

The predicted forecast of MMR in East Africa in 2030 is 168 deaths per 100,000 births (Figure 11).

### 3.3. Central Africa

**Figure 12:**
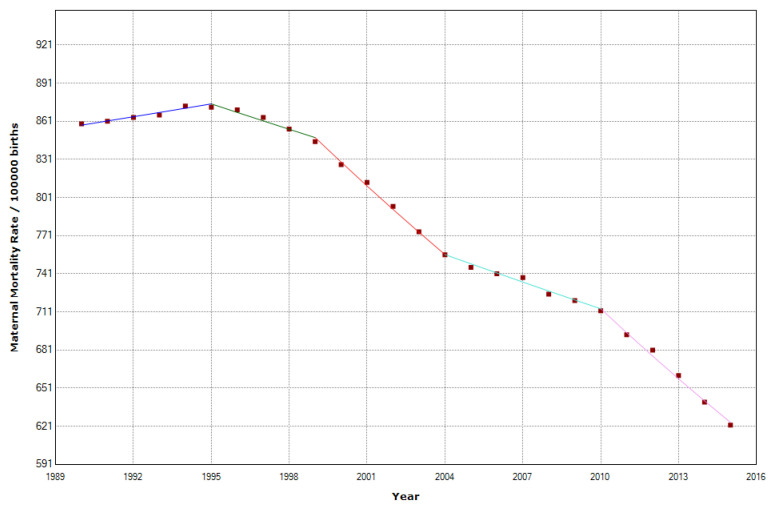
Maternal mortality trends in Central Africa (1990–2015) indicating joinpoints at the transitions between colored lines.

In Central Africa, there was a reduction of 33.5% in MMR, which moved from 708 in 1990 to 471 in 2015. The joinpoint regression shows that in the Central African Region, there were four joinpoints (Table 3). From 1990 to 1995, maternal mortality increased by 0.4% per year; from 1995 to 1999, the MMR began to decline with an APC of −0.8%. After that, during 1999–2004, the APC accelerated with an APC of −2.3%. The decline slowed in 2004–2010 with an APC of −1% and accelerated again in 2010–2015 with an APC of −2.6% (Figure 12).

**Figure 13:**
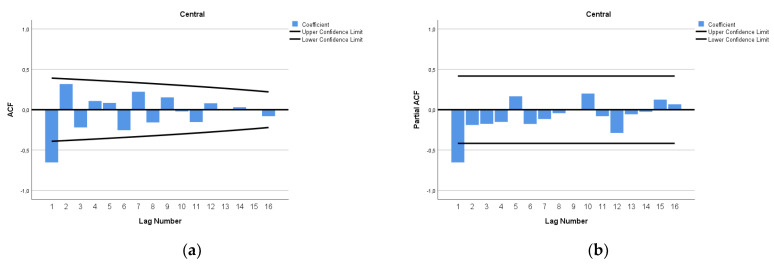
Maternal mortality in Central Africa (**a**) autocorrelation (ACF) (**b**) and partial autocorrelation (PACF).

The ARIMA model of MMR in Central Africa was (1,2,0). In Figure 13, we present the ACF and PACF of the temporal series of MMR in Central Africa. In the ARIMA model, the value of the stationary R-squared was 0.145, an R-squared of 0.998, and the normalized Bayesian Information Criterion (BIC) was 2.925.

**Figure 14:**
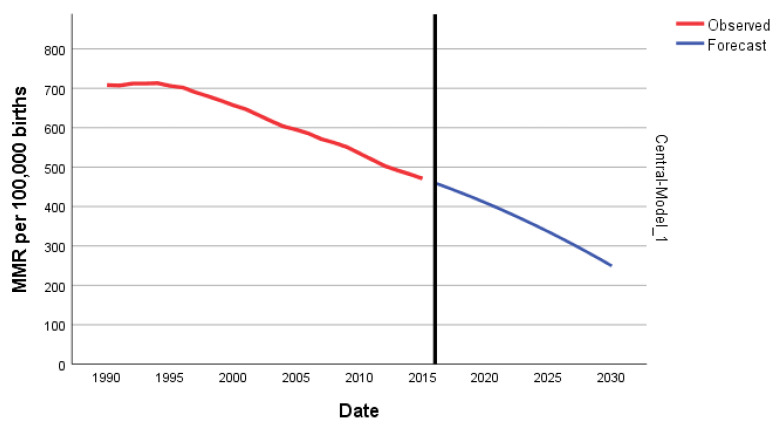
Evolution and forecasting of MMR in Central Africa.

The predicted forecast of MMR in Central Africa in 2030 is 249 deaths per 100,000 births (Figure 14).

### 3.4. South Africa

**Figure 15:**
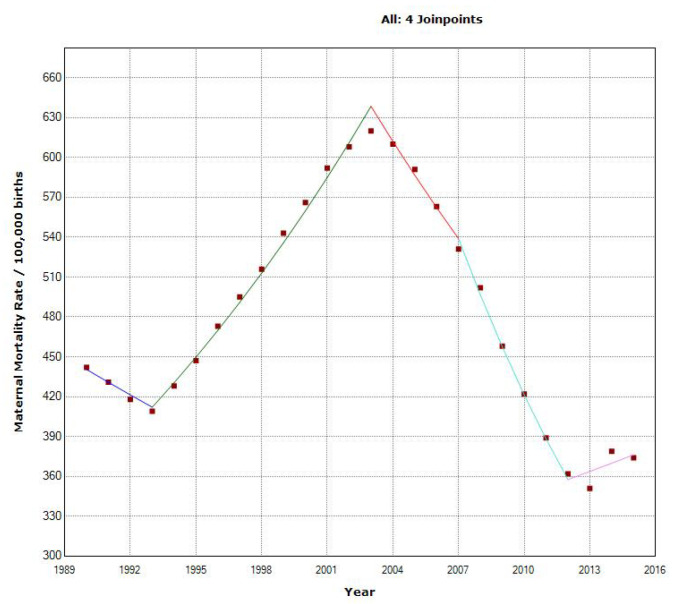
Maternal mortality trends in the South Africa region (1990–2015) indicating joinpoints at the transitions between colored lines.

The overall MMR decreased in the South African Region from 442 to 374 per 100,000. We recorded a statistically significant decrease of −0.6% in the MMR, with four joinpoints in 1993, 2003, 2007, and 2012 (Figure 15). In 1990–1993, MMR decreased with an APC of −2.2%, then in the second period, 1993–2003, MMR increased, with an APC of 4.5%. Finally, in 2003–2007, it returned to an APC of −4.1%, and the period 2007–2012 had a high APC decrease of −7.9%. This is the highest regional decrease detected in this study.

**Figure 16:**
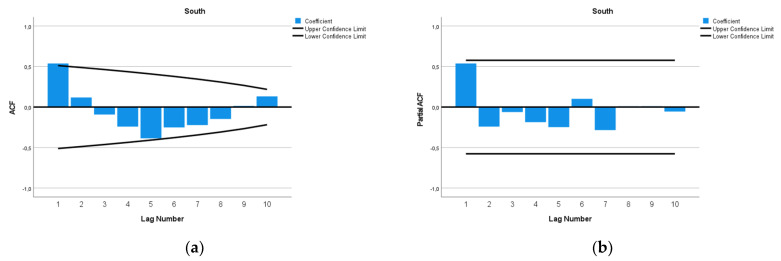
Maternal mortality in South Africa (**a**) autocorrelation (ACF) (**b**) and partial autocorrelation (PACF).

The ARIMA model of MMR in the South Africa region was ARIMA (0,1,1). This study analyzed the maternal mortality rates (MMRs) in South Africa from 1990 to 2015. The data exhibited a distinct pattern, with MMR increasing from 442 per 100,000 live births in 1990 to a peak of 620 per 100,000 live births in 2003. After 2003, the MMR decreased, reaching 374 per 100,000 live births in 2015. Given the observed inverted ‘V’ pattern in the data, with a vertex in 2003, we decided to fit ARIMA models using data from 2003 onwards. The decision to make this estimation was grounded on the notable structural change observed in the data around 2003, indicating a shift in the dynamics of the MMR. We used a square root transformation of the data for a better fit. In Figure 16, we present the ACF and PACF of the temporal series of MMR in South Africa. In the ARIMA model, the value of the stationary R-squared was 0.525, an R-squared of 0.966, and the normalized Bayesian Information Criterion (BIC) was 6.015.

**Figure 17:**
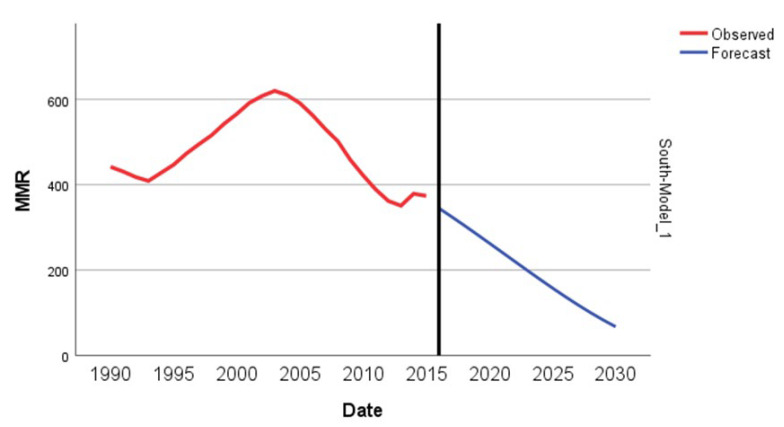
Evolution and forecasting of MMR in South Africa.

The predicted forecast of MMR in the South Africa region in 2030 is 67 deaths per 100,000 births (Figure 17).

### 3.5. West Africa

**Figure 18:**
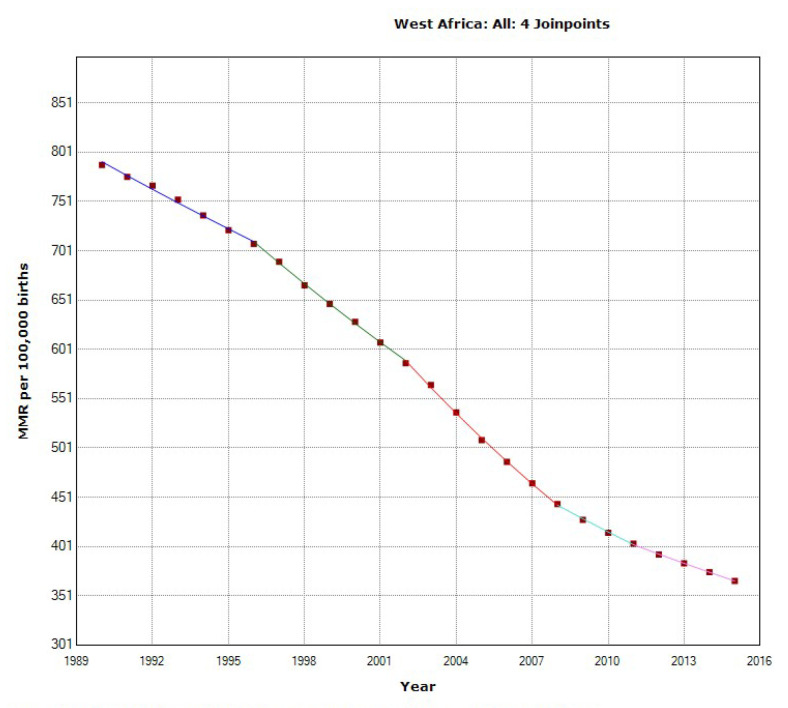
Maternal mortality trends in the West Africa region (1990–2015) indicating join points at the transitions between colored lines.

In the West Africa region, the overall MMR decreased by 53.6% from 788 to 366 during 1995–2015. We documented a statistically significant decrease in the MMR, with an APC of −2.6%, with four joinpoints in 1996, 2002, 2008, and 2011 (Figure 18).

**Figure 19:**
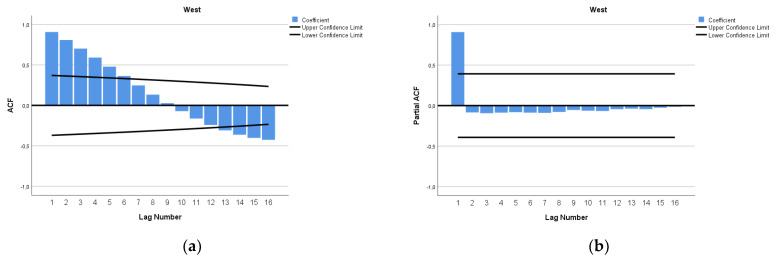
Maternal mortality in West Africa (**a**) autocorrelation (ACF) (**b**) and partial autocorrelation (PACF).

The ARIMA model for forecasting MMR in West Africa was ARIMA (1,2,3). In Figure 19, we present the ACF and PACF of the temporal series of MMR in North Africa. In the ARIMA model, the value of the stationary R-squared was 0.226, an R-squared of 1, and the normalized Bayesian Information Criterion (BIC) was 2.952.

**Figure 20:**
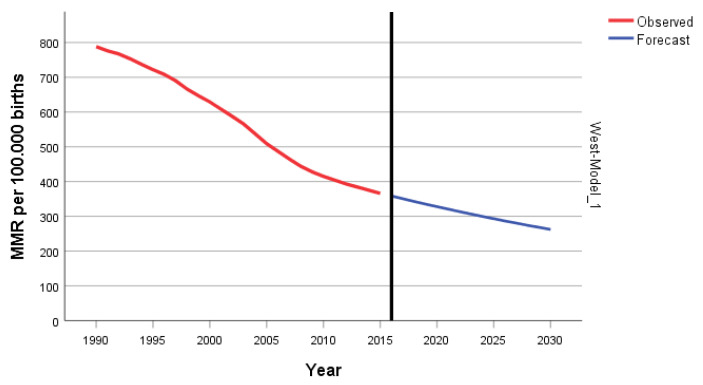
ARIMA predictions and historical trends of MMR in West Africa.

The predicted forecast of MMR in the West Africa region in 2030 is 262 deaths per 100,000 births (Figure 20).

### 3.6. Holt Forecasting

**Figure 21:**
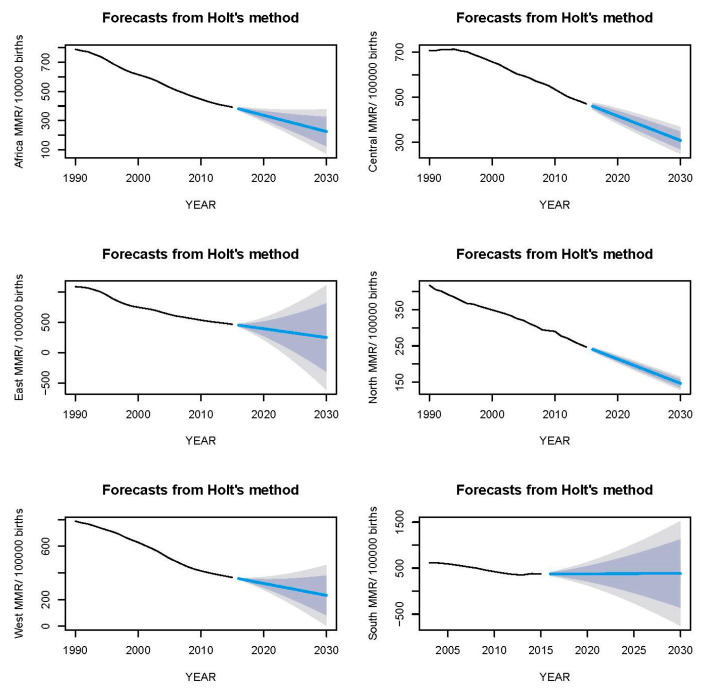
Comparative analysis and projection of maternal mortality rates in Africa and its regions utilizing the Holt method. (The blue shaded area signifies the 80% CI and the grey represents the 95% CI).

The Holt estimations are similar to the ARIMA’s (Table 4). The Holt Exponential Smoothing method forecast of MMR in Africa for 2030 is 225 deaths per 100,000 births (Figure 21), accompanied by a mean absolute percentage error (MAPE) of 0.557; in Central Africa, an MMR of 308 and a MAPE of 0.425; in East Africa, an MMR of 248 and a MAPE of 0.766; in North Africa, an MMR of 146 and a MAPE of 0.701; in West Africa, an MMR of 231 and a MAPE of 0.394; and in South Africa, an MMR of 309 and a MAPE of 2.361.

### 3.7. Machine Learning Forecasting

**Figure 22:**
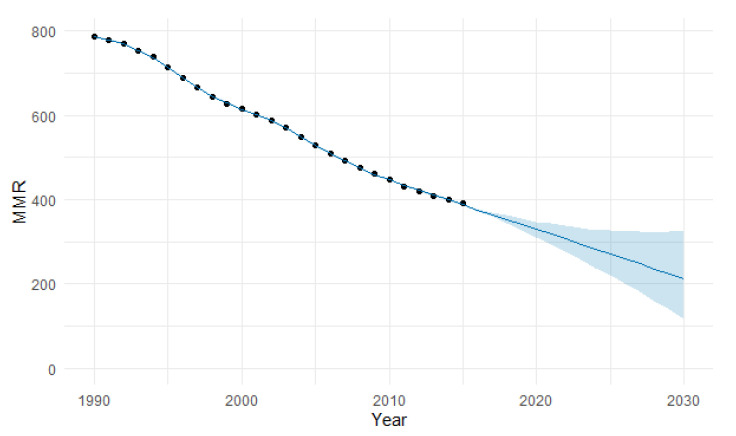
Machine learning projections of MMR in Africa using the PFM method, including 95% uncertainty intervals (UI). (The blue shaded area signifies the 95% UI).

The PFM estimation of the MMR in Africa for 2030 was 213 per 100,000 births, with a 95% uncertainty interval (UI) between 110 and 314 (Table 4). The model had a MAPE of 3.91% (Table 5 and Figure 22). This estimation is lower than the ARIMA 182.

**Figure 23:**
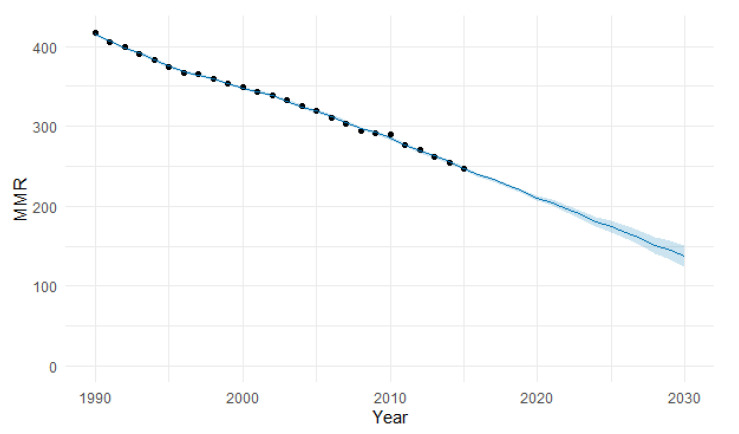
Machine learning projections of MMR in North Africa using the PFM method, including 95% uncertainty intervals. (The blue shaded area signifies the 95% UI).

The PFM estimation of the MMR in North Africa for 2030 was 138 deaths per 100,000 births (95% UI 124–151) (Figure 23), with a MAPE of 3.91% (Table 4). The machine learning estimation was slightly lower than the ARIMA and the Holt method estimations, with MMRs of 161 and 146, respectively.

**Figure 24:**
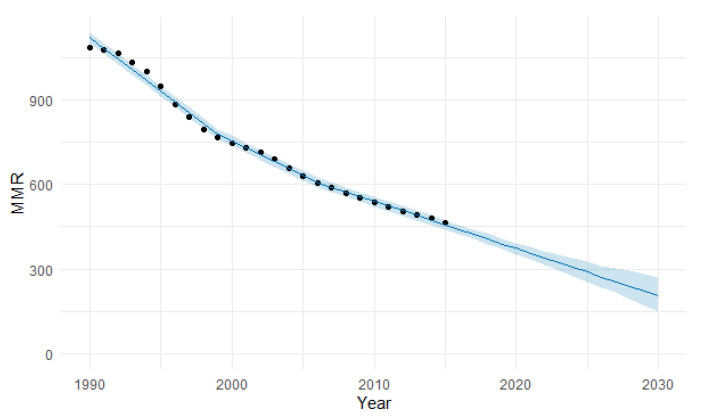
Machine learning projections of MMR in East Africa using the PFM method, including 95% uncertainty intervals. (The blue shaded area signifies the 95% UI).

The PFM estimation of the MMR in East Africa in 2030 was 204 (95% UI 147–270) with a MAPE of 5.78% (Table 4 and Figure 24). This estimation is lower than the ARIMA and Holt methods, with 270 and 248 deaths per 100,000 births.

**Figure 25:**
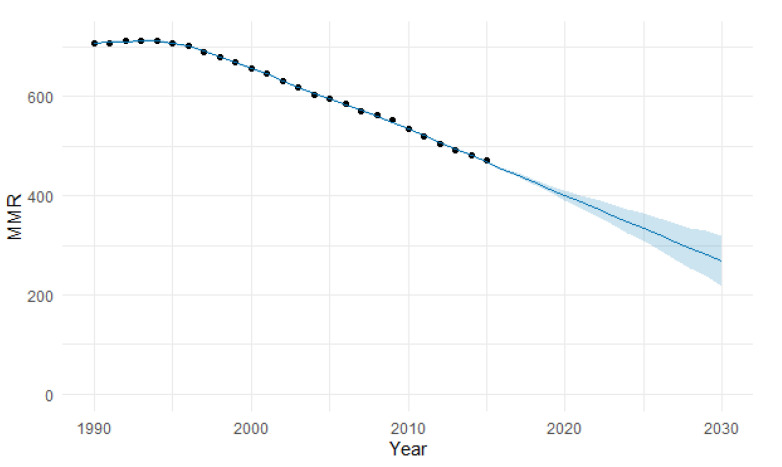
Machine learning projections of MMR in Central Africa using the PFM method, including 95% uncertainty intervals. (The blue shaded area signifies the 95% UI).

The PFM estimation of the MMR in Central Africa in 2030 is 267 (95% UI 218–320) with a MAPE of 2.38% (Table 4 and Figure 25). This estimation is lower than that of ARIMA, 302 deaths per 100,000 births, and the Holt method, 308 deaths per 100,000 births.

**Figure 26:**
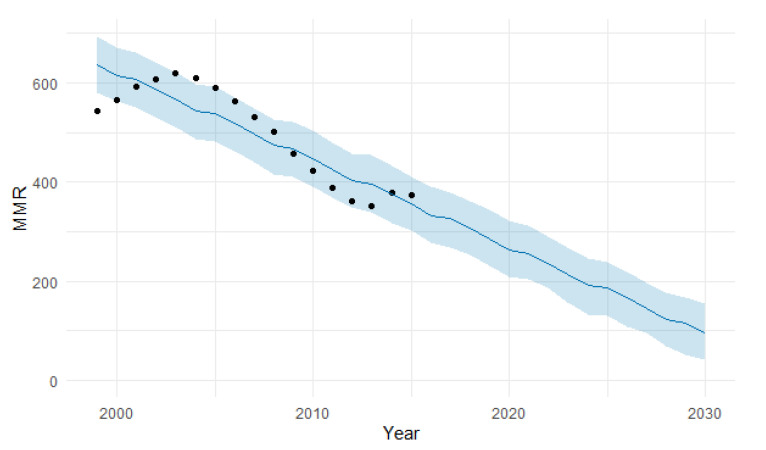
Machine learning projections of MMR in South Africa using the PFM method, including 95% uncertainty intervals. (The blue shaded area signifies the 95% UI).

To compute the PFM estimation, we could not use the whole temporal series of South Africa because the MAPE was very high, 62%. Due to that, only the data from 2003 onward were used. The PFM estimation of the MMR in South Africa in 2030 is 95 per 100,000 births (95% UI 41–153) with a MAPE of 28% (Table 4 and Figure 26). This estimation is lower than that of ARIMA, with 156 deaths per 100,000 births, and very far away from that of the Holt method, with an estimated MMR of 382 per 100,000 births.

**Figure 27:**
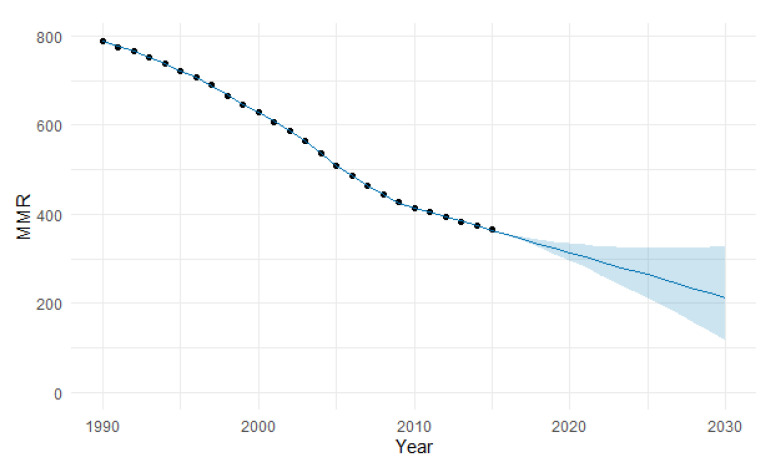
Machine learning projections of MMR in West Africa using the PFM method, including 95% uncertainty intervals. (The blue shaded area signifies the 95% UI).

The PFM estimation of the MMR in West Africa for 2030 is 262 deaths per 100,000 births with a 95% uncertainty interval (UI) between 116 and 325 (Figure 27), with a MAPE of 9.15% (Table 4). The machine learning estimation was similar to those of the ARIMA, with 262 deaths per 100,000 births, and the Holt method, with an MMR of 231.

## 4. Discussion

Africa is the region with the highest maternal mortality rate in the world, but the region lacks accurate data. Researchers have employed various methods to estimate maternal mortality in Africa [49]. However, the heterogeneity of these methods affects the results and makes it difficult to compare them.

Reproductive age mortality survey (RAMOS) studies combining data from institutional records and communities produced the most reliable national maternal mortality estimates. Many Sub-Saharan African countries rely on various surveys and census methods, such as Multiple Indicator Cluster Surveys, Demographic and Health Surveys, and population censuses, to estimate their MMR [50].

While our analysis has offered valuable insights into the trends, projections, and regional disparities of maternal mortality rates (MMRs) in Africa from 1990 to 2030 using an ARIMA forecasting approach, it is essential to acknowledge some of its limitations. Primarily, this study relied exclusively on data from the World Bank database, which, though extensively used and recognized for its reliability, may not wholly encapsulate the intricacies and nuances of MMRs across the diverse African continent. The reliability and completeness of the World Bank’s MMR data could potentially vary across countries and regions within Africa due to the disparities in data collection, reporting standards, and healthcare infrastructure. Consequently, our analysis might be subject to some degree of imprecision.

Future research could enhance the robustness and comprehensiveness of such analysis by incorporating data from multiple sources. The World Health Organization, for instance, maintains comprehensive health-related data sets that could be integrated into future analyses. Similarly, national health surveys often provide detailed and locally specific data that could capture certain nuances missed by more extensive international databases. Furthermore, data from health-focused non-governmental organizations (NGOs) operating in Africa could offer unique insights into grassroots realities and fill potential gaps in national reporting. By integrating these diverse sources, future studies could offer a more holistic and nuanced understanding of African MMRs. Such multi-source analysis might also help mitigate potential regional or national biases in the data and help to build a more comprehensive and accurate understanding of MMR trends and projections across Africa.

The ARIMA model, widely recognized for its ability to manage intricate datasets characterized by autocorrelation and non-stationarity, has found broad applications in epidemiological research. Its adaptability and precision have been documented in multiple studies. For example, a research study implemented the ARIMA model to predict future trends in measles immunization coverage [35]. Using time-series data from January 2014 to December 2018, the investigators established the best-fit ARIMA (0,1,0) model to predict immunization coverage for the subsequent 36 months. The successful forecasts from this study underscore the utility of ARIMA in making informed immunization policies and resource allocations.

Moreover, the ARIMA model has demonstrated utility in predicting the trajectory of infectious diseases, such as influenza [36]. A Seasonal ARIMA (SARIMA) model was adopted in one study to simulate and project the influenza epidemic dynamics in Ukraine, facilitating the planning of efficient anti-epidemic measures. This demonstrates ARIMA’s potential in steering strategies for infectious disease control through reliable forecasting. Furthermore, ARIMA modeling has been deployed to forecast maternal death rates in the context of COVID-19 vaccinations [37]. The research utilized the ARIMA model to compare the anticipated number of maternal deaths for 2021 in Mexico, with and without vaccinating pregnant women against COVID-19. The forecasts indicated a substantial reduction in maternal deaths with full vaccination coverage, emphasizing ARIMA’s pivotal role in directing healthcare interventions and policies. These varied ARIMA usages in epidemiological studies underline its proficiency in delivering solid, trustworthy forecasts and shaping public health strategies and interventions. Within this research, we aim to apply this prowess to dissect the trends, projections, and regional disparities of maternal mortality in Africa from 1990 to 2030.

The overall decline in maternal mortality across Africa from 1990 to 2015 is a positive outcome, reflecting global and regional efforts to address maternal health issues. However, the current MMR rates remain alarmingly high, and regional disparities persist. The annual decline of maternal mortality in Africa by 2.6% over the study period is promising. However, the change is less than the 5.5% annual decrease necessary to achieve the SDG target. This outcome underscores the need for accelerated efforts and innovative interventions in maternal healthcare.

The global African maternal mortality rate presented in our study, 392 per 100,000, is within the range of a meta-analysis conducted with studies published in Africa with 496 per 100,000 births (95% CI 216–776) [51]. We have estimated using the ARIMA, Holt, and machine learning methods. MAPE measures the prediction accuracy of a forecasting method in statistics. It expresses the average absolute percent difference between actual and predicted values, with lower values indicating better model accuracy. The projections computed by the ARIMA, PFM, and Holt methods are similar, and their confidence intervals overlap.

Research has shown that PFM outperforms traditional models like ARIMA and SARIMA [45,52]. Additionally, PFM has a significantly faster training time, about ten times quicker than ARIMA [52].

This study’s 25-year dataset provides a robust basis for projecting trends 15 years into the future, capitalizing on the ARIMA model’s ability to detect long-term patterns [53]. The limited time points, albeit fewer than recommendations of 50 to 100 observations [54,55], reflect an intentional balance to prioritize data relevance over volume, given the potential for substantial shifts and resulting noise in the underlying process. Starting from an earlier year, like 1965, would incorporate a period of effective decolonization in Africa, which could compromise future predictions. The lack of seasonality in the data aids model fitting, although multiple inflection points detected by joinpoint analysis signify non-stationarity, requiring careful application of the ARIMA model. The 15-year forecast period comes with compounded errors and increased uncertainty, exacerbated by the potential for unanticipated structural changes. Such factors emphasize the need for a cautious interpretation of long-term forecasts, periodic re-evaluations, and confidence interval incorporation to gauge uncertainty [56] Additionally, integrating techniques like scenario planning or expert forecasting could enhance ARIMA’s inherent limitations in addressing potential future events or changes.

It is essential to consider socioeconomic and healthcare factors when understanding maternal mortality rate (MMR) trends in African regions. Socioeconomic factors such as income, education, and employment directly or indirectly affect health outcomes [57,58,59,60,61,62,63,64,65,66,67]. Healthcare factors, on the other hand, include health policies, healthcare infrastructure, accessibility, and quality of care [68,69,70,71,72,73,74,75,76,77].

For North Africa, which has consistently lower MMR, factors contributing to this trend could include better healthcare infrastructure, the availability of skilled healthcare providers, and higher rates of female education and economic participation [78,79,80]. Access to antenatal care and emergency obstetric services is likely more prevalent in this region than in Sub-Saharan Africa [81]. The MMR remains relatively high compared to global standards, indicating room for improvements in socioeconomic conditions and healthcare services [82,83,84].

Over the past twenty years, no other region has experienced as many significant military confrontations as Sub-Saharan Africa (SSA), which underwent 13 wars from 1990 to 2015 [85]. This heightened state of conflict has detrimentally impacted the region’s healthcare infrastructure.

In East Africa, a significant decline in MMR could be associated with various factors. From 1990 to 2015, the East African region, particularly the Horn of Africa, experienced a high frequency of conflicts, which may have significantly impacted maternal mortality rates. However, there has been a noticeable reduction in conflicts in recent years, indicating a move toward greater regional peace and stability [86,87]. Initiatives to improve the quality of maternal healthcare services, enhance access to education, and alleviate poverty may have played a key role [88,89,90,91]. However, the MMR remains relatively high, suggesting that challenges such as health system weaknesses, the low socioeconomic status of women, and limited access to healthcare services are still prevalent [92,93,94,95,96,97,98,99].

The Central African region, despite some improvements, still has a high MMR. Our analysis revealed a downward trend in MMR over the 25 years, suggesting an overall improvement in maternal health outcomes in the region. The initial rise in MMR from 1990 to 1995 could be attributed to socioeconomic challenges and political upheavals [100]. Subsequent marginal decrease until 1999 could result from early efforts to bolster regional healthcare systems, especially those coordinated by international health organizations. The accelerated decline in MMR between 1999 and 2004 coincided with substantial efforts to enhance healthcare access, quality of care, and overall socioeconomic conditions. However, the slowed rate of decrease from 2004 to 2010 may indicate unaddressed obstacles, possibly persistent healthcare inequities, socio-political issues, and external shocks like economic crises or disease outbreaks.

The renewal of global attention to maternal health after 2010, exemplified by initiatives such as the United Nations’ Millennium Development Goals, seems to have reinvigorated the MMR decline. Therefore, it is paramount to prioritize interventions addressing systemic and structural issues, socioeconomic development, and strengthening healthcare services [101,102]. Continued investments, particularly in maternal health, will be integral to sustain and further accelerate the reduction in MMR in the region. Our findings underscore the importance of a multi-pronged and sustained approach to addressing maternal mortality in Central Africa [103,104]. Moreover, this region may still grapple with challenges such as low levels of female education, high fertility rates, poor access to maternal healthcare services, and poverty.

In South Africa, the fluctuating MMR trend could be influenced by numerous factors. The increase until 2003 might reflect the HIV/AIDS epidemic’s impact, as the region has a high prevalence [105]. The subsequent decrease could be due to extensive HIV/AIDS interventions, including the provision of antiretroviral therapy. Despite this, the MMR remains relatively high, which could be attributed to persistent socioeconomic inequalities, lapses in healthcare services, and other health challenges like non-communicable diseases.

The West African region also saw a significant decrease in MMR, but the rates remain high. Challenges here may include poor access to quality maternal health services, high fertility rates, low female education levels, socio-cultural practices, and poverty [106]. Challenges related to infrastructure, difficulties with human resources, limited access to vital medications, subpar healthcare quality, superstitions, and cultural beliefs contribute to MMR [107].

These explanations should be considered hypotheses that require further exploration. For instance, examining variables such as gross domestic product, female education level, fertility rate, healthcare spending, HIV/AIDS prevalence, and others in each region could provide more concrete insights [108]. However, these interpretations can guide policymakers to focus on socioeconomic development and comprehensive healthcare system strengthening as crucial strategies for further reducing MMR.

Since the 2007 economic crisis, we have observed a decline in maternal mortality in Africa. We noted the diminishing reduction in the Northern, Central, and Western regions. The economic crisis impacted the East region but left the South region unscathed. As an integral component of the SDGs in 2015, the United Nations aimed to reduce the MMR to 70 out of every 100,000 live births by 2030. According to our forecasting, no region will reach the target. Reducing maternal deaths is among the most challenging SDG targets because Africa would need an 86 percent reduction in MMR, which is unrealistic given the present rate of decline [48].

Institutional delivery, which refers to childbirth facilitated by trained healthcare professionals following safe and sterile protocols, is crucial to maternal health. Enhancing maternal understanding, perspective, and the utilization of institutional delivery contributes significantly to decreasing maternal mortality and morbidity rates [109]. In Africa, where many regions face considerable challenges in emergency obstetric care, the emphasis on institutional delivery becomes even more critical because there are widespread systemic issues in emergency obstetric care across Africa.

In South Africa, several studies have shown that in regions grappling with significant challenges in emergency obstetric care, the focus on institutional delivery is paramount due to prevalent systemic issues. Overburdened healthcare providers, notable personnel shortages, and problems with service delivery and infrastructure typify these issues. Compounded by limited access to vital resources such as operating theaters, intensive care units, and reliable emergency transport services, these challenges significantly amplify the risk of severe maternal outcomes. These circumstances underline systemic weaknesses, emphasizing the need for improvements in service coordination, staffing, performance indicators, and overall funding [110]. Despite national policy guidelines advocating for decentralizing emergency obstetric care to Community Health Centers, services were centralized in hospitals to ensure patient safety [111]. The use of comprehensive emergency obstetric and newborn care is related to waiting time for service and women’s educational level [111,112]. This could suggest that improving women’s education may increase the utilization of comprehensive emergency obstetric services. Therefore, future assessments of emergency obstetric care availability should consider facility opening hours, capacity, staffing, and the proven performance of essential functions rather than merely the venue of service provision. On the other hand, enhancing male education, improving wealth distribution, ensuring access to health facilities, promoting post-natal care, and improving the quality of antenatal and maternity care services could significantly increase the utilization of skilled delivery services in rural areas [50]. In Uganda, unmarried and married youth who frequently attend antenatal care visits and attain higher education demonstrate increased utilization of health facilities during childbirth [50]. Conversely, health facility usage negatively correlates with higher parity, rural residence, and employment in the agriculture sector. Promoting regular antenatal care attendance is essential to encourage youth use of health facilities, particularly for those with multiple children residing in rural areas. Policies should focus on improving access to mass media, enhancing youth education, and addressing economic disparities. An important area not covered in our study is pregnancy-related mortality in the extended postpartum, pointing out the lack of coordination between primary healthcare and obstetric services [113,114].

The African Union Summit in 2019 decided to increase domestic health resources [115]. African countries are gradually increasing domestic health investments, with 35 of 55 African Union Member States (more than 64%) increasing the percentage of their GDP invested in health over the previous fiscal year. Out of the 55 member states in the African Union (AU), just two countries have successfully achieved Africa’s objective of allocating a minimum of 15% of their government budget toward healthcare. However, these countries still fail to reach the recommended threshold of US$86.30 per person, which is necessary to offer essential health services [115,116,117]. Africa must financially commit to its health. The 2014 African Ministries of Health conference in Luanda emphasized the importance of improved transportation, communication technologies, effective community participation, and men’s involvement in improving maternal health. They also emphasized the importance of investing in the development of human resources for health and the importance of adolescent health in the survival of mothers [18].

The impact of the COVID-19 pandemic appears to pose substantial challenges to maternal health, particularly in Africa. Although detailed data are forthcoming, early indications suggest that the pandemic has significantly impacted maternal and child healthcare [118]. The limited availability of skilled health professionals and medical equipment, service disruptions, and an increased reluctance among women to use available healthcare facilities contribute to this impact [119,120]. Despite the absence of specific statistics on the pandemic’s effects on maternal mortality, studies conducted in countries like Guinea, Nigeria, Tanzania, and Uganda hint at a decline in critical maternal and child health services, particularly in antenatal, intrapartum, and post-natal care [121] This decline could potentially hinder progress toward reducing maternal mortality rates, posing a threat to achieving the related Sustainable Development Goal (SDG).

## 5. Policy Implications

The study findings highlight the need to prioritize reducing maternal mortality in Africa, particularly Central Africa. Policymakers, international organizations, and NGOs should focus on region-specific strategies to address disparities in healthcare systems, socioeconomic contexts, and policy environments. Maternal health interventions should include broader socioeconomic determinants beyond the medical domain. These could include policies to increase literacy rates, improve women’s status, and enhance income. Particular attention should be given to protecting maternal health during global crises such as economic downturns. There is also a pressing need to improve the data collection system to ensure comprehensive and regular data collection and validation, which are crucial for effective policymaking. Our study emphasizes that achieving the SDGs for maternal health in Africa requires concerted efforts from all stakeholders. Such efforts should focus on direct health interventions and improving the broader socioeconomic conditions significantly influencing maternal health outcomes.

## 6. Conclusions

Over the study period, maternal mortality has decreased in Africa by an average APC of 2.6%. All regions showed a significant downward trend, with the sharpest decreases in East Africa. Only the South and North African regions are close to the United Nations’ sustainable development goals for maternal mortality. The remaining Sub-Saharan African regions are still far from achieving the goals. These results show the need to develop regional policies further to decrease maternal mortality in Africa. Our study concludes that despite a decreasing trend, the MMR in Africa remains high, with significant disparities across different regions. The ARIMA model forecasts reveal that no region in Africa will likely reach the SDG target in 2030, indicating the urgent need for amplified efforts, especially in Central Africa. Moreover, socioeconomic contexts and global events significantly influence maternal mortality rates, impacting different regions’ progress pace. Therefore, strategies to reduce MMR should also focus on improving socioeconomic conditions, especially during global crises like economic downturns. Although our research provides valuable insights into MMR trends and projections, it does not delve into the specific interventions needed to accelerate MMR reduction. Future research should focus on developing region-specific intervention strategies based on each region’s unique socioeconomic and healthcare context.

## Figures and Tables

**Table 1:** Joinpoint analysis of maternal mortality rates in Africa, 1990–2015.

Periods	Years	APC (95% CI)	*p*
Total Period	1990–2015	−2.6 (−2.7; −2.5)	<0.001
Period 1	1990–1996	−1.4 (−1.6; −1.2)	<0.001
Period 2	1996–2001	−2.0 (−2.4; −1.7)	<0.001
Period 3	2001–2007	−3.5 (−3.8; −3.3)	<0.001
Period 4	2007–2015	−2.7 (−2.8; −2.6)	<0.001

**Table 2:** Parameters of the ARIMA stationary R-squared and normalized Bayesian Information Criterion (BIC) of ARIMA models of maternal mortality by region, 1990–2015.

Region	Models	Stationary R-Squared	BIC
Africa	ARIMA (1,1,1).	0.277	3.662
North	ARIMA (1,2,0).	0.289	3.037
East	ARIMA (1,3,0)	0.148	4.696
Central *	ARIMA (1,2,0).	0.145	2.925
South	ARIMA (0,1,1).	0.525	6.015.
West	ARIMA (1,2,3).	0.226	2.952

* 2003–2015.

**Table 3:** Joinpoint analysis of maternal mortality rates in Africa, 1990–2015.

Periods	Years	APC (95% CI)	*p*
North			
Total Period	1990–2015	−1.9 (−2.0; −1.8)	<0.001
Period 1	1990–1996	−2.0 (−2.1; −1.8)	<0.001
Period 2	1996–2003	−1.4 (−1.5; −1.3)	<0.001
Period 3	2003–2007	−2.5 (−2.8; −2.1)	<0.001
Period 4	2007–2010	−1.7 (−2.6; −0.8)	0.001
Period 5	2010–2015	−3.0 (−3.2; −2.9)	<0.001
East			
Total Period	1990–2015	−3.6 (−3.8; −3.4)	<0.001
Period 1	1990–1993	−1.3 (−1.5; −1.1)	<0.001
Period 2	1993–2001	−2.6 (−2.7; −2.6)	<0.001
Period 3	2001–2004	−3.2 (−3.7; −2.8)	<0.001
Period 4	2004–2009	−5.0 (−5.1; −4.9)	<0.001
Period 5	2009–2015	−4.5 (−4.6; −4.4)	<0.001
Central			
Total Period	1990–2015	−1.3 (−1.5; −1.1)	<0.001
Period 1	1990–1995	0.4 (0.1; 0.6)	0.005
Period 2	1995–1999	−0.8 (−1.3; −0.2)	0.009
Period 3	1999–2004	−2.3 (−2.6; −1.9)	<0.001
Period 4	2004–2010	−1.0 (−1.2; −0.7)	<0.001
Period 5	2010–2015	−2.6 (−2.9; −2.4)	<0.001
South			
Total Period	1990–2015	−2.6 (−2.8; −2.4)	<0.001
Period 1	1990–1994	−1.4 (−1.8.; −0.9)	<0.001
Period 2	1994–2003	−1.9 (−2.0; −1.7)	<0.001
Period 3	2003–2012	−3.6 (−3.7; −3.5)	<0.001
Period 4	2013–2015	−2.0 (−3.2; −0.8)	0.004
West			
Total Period	1990–2015	−3.3 (−3.5; −3.2)	<0.001
Period 1	1990–1996	−1.8 (−1.9.; −1.6)	<0.001
Period 2	1996–2002	−3.1 (−3.3; −2.9)	<0.001
Period 3	2002–2008	−4.7 (−4.9; −4.5)	<0.001
Period 4	2008–2011	−3.1 (−4.0; −2.2)	<0.001
Period 5	2011–2015	−2.4 (−2.7; −2.1)	<0.001

**Table 4:** Comparison of ARIMA, Prophet Model, and Holt Regional Projections of MMR in Africa for 2030 deaths/100,000 births.

Region	ARIMA	Prophet Model	Holt
Africa	182 (95% CI 93–271)	213 (95% UI 110–314)	225 (95% CI 72–377)
North	161 (95% CI 88–273)	138 (95% UI 124–151)	146 (95% CI 129–164)
East	270 (95% CI 96–623)	204 (95% UI 147–270)	248 (95% CI 0–1110) *
Central	302 (95% CI 214–414)	267 (95% UI 218–320)	308 (95% CI 248–367)
South **	156 (95% CI 48–310)	95 (95% UI 41–153)	382 (95% CI 0–1518) *
West	243 (95% CI 197–297)	262 (95% UI 116–325)	231 (95% CI 3–458)

* Lower limit of the 95% CI set to 0; ** 2003–2015.

**Table 5:** Mean absolute percentage error (MAPE), root mean squared error (RMSE), and mean absolute error (MAE) of the Prophet Forecasting Model for MMR in Africa, 1990–2015.

Region	MAPE	RMSE	MAE
Africa	3.9128	8.9247	15.8442
North	0.9653	3.6545	2.6770
East	5.7883	31.6667	28.2150
Central	2.3891	12.3689	11.8040
South *	28.5623	108.6146	107.2973
West	9.1557	39.1982	34.8333

* 2003–2015.

## Data Availability

Data are available online at the World Bank data bank https://databank.worldbank.org/source/population-estimates-and-projections# (accessed on 28 June 2023).

## References

[1] Bagade T., Chojenta C., Harris M., Oldmeadow C., Loxton D. (2022). The Human Right to Safely Give Birth: Data from 193 Countries Show That Gender Equality Does Affect Maternal Mortality. BMC Pregnancy Childbirth.

[2] Bueno de Mesquita J., Kismödi E. (2012). Maternal Mortality and Human Rights: Landmark Decision by United Nations Human Rights Body. Bull. World Health Organ.

[3] World Health Organization (2023). Trends in Maternal Mortality 2000 to 2020: Estimates by WHO, UNICEF, UNFPA, World Bank Group and UNDESA/Population Division.

[4] ICD-11 Reference Guide. https://icdcdn.who.int/icd11referenceguide/en/html/index.html.

[5] Fleszar L.G., Bryant A.S., Johnson C.O., Blacker B.F., Aravkin A., Baumann M., Dwyer-Lindgren L., Kelly Y.O., Maass K., Zheng P. (2023). Trends in State-Level Maternal Mortality by Racial and Ethnic Group in the United States. JAMA.

[6] Abbasi J. (2023). US Maternal Mortality Is Unacceptably High, Unequal, and Getting Worse—What Can Be Done About It?. JAMA.

[7] Say L., Chou D., Gemmill A., Tunçalp Ö., Moller A.-B., Daniels J., Gülmezoglu A.M., Temmerman M., Alkema L. (2014). Global Causes of Maternal Death: A WHO Systematic Analysis. Lancet Glob Health.

[8] WHO (2019). Trends in Maternal Mortality 2000 to 2017: Estimates by WHO, UNICEF, UNFPA, World Bank Group and the United Nations Population Division.

[9] Khan K.S., Wojdyla D., Say L., Gülmezoglu A.M., Van Look P.F. (2006). WHO Analysis of Causes of Maternal Death: A Systematic Review. Lancet.

[10] Bhutta Z.A., Das J.K., Bahl R., Lawn J.E., Salam R.A., Paul V.K., Sankar M.J., Blencowe H., Rizvi A., Chou V.B. (2014). Lancet Newborn Interventions Review Group; Lancet Every Newborn Study Group. Can Available Interventions End Preventable Deaths in Mothers, Newborn Babies, and Stillbirths, and at What Cost?. Lancet.

[11] Gabrysch S., Campbell O.M. (2009). Still Too Far to Walk: Literature Review of the Determinants of Delivery Service Use. BMC Pregnancy Childbirth.

[12] United Nations General Assembly (2015). Transforming Our World: The 2030 Agenda for Sustainable Development United Nations.(A/RES/70/1).

[13] Kruk M.E., Gage A.D., Arsenault C., Jordan K., Leslie H.H., Roder-DeWan S., Adeyi O., Barker P., Daelmans B., Doubova S.V. (2018). High-Quality Health Systems in the Sustainable Development Goals Era: Time for a Revolution. Lancet Glob Health.

[14] Van den Broek N.R., Falconer A.D. (2011). Maternal Mortality and Millennium Development Goal 5. Br. Med. Bull..

[15] United Nations Millennium Development Goals. https://www.un.org/millenniumgoals/.

[16] WHO, UNICEF, UNFPA, W.B.G., U.N.P.D (2015). Trends in Maternal Mortality: 1990 to 2015 Estimates.

[17] Temmerman M., Khosla R., Bhutta Z.A., Bustreo F. (2015). Towards a New Global Strategy for Women’s, Children’s and Adolescents’ Health. BMJ.

[18] African Union, WHO (2014). First Meeting of African Ministers of Health Jointly Convened by the AUC and WHO.

[19] World Bank Data Bank Africa Development Indicators. http://data.worldbank.org/data-catalog/africa-development-indicators.

[20] GBD 2015 Maternal Mortality Collaborators (2016). Global, Regional, and National Levels of Maternal Mortality, 1990–2015: A Systematic Analysis for the Global Burden of Disease Study 2015. Lancet.

[21] WHO (2009). Monitoring Emergency Obstetric Care: A Handbook.

[22] Gabrysch S., Simushi V., Campbell O.M.R. (2011). Availability and Distribution of, and Geographic Access to Emergency Obstetric Care in Zambia. Int. J. Gynecol. Obstet..

[23] Admasu K., Haile-Mariam A., Bailey P. (2011). Indicators for Availability, Utilization, and Quality of Emergency Obstetric Care in Ethiopia, 2008. Int. J. Gynaecol. Obstet..

[24] Gabrysch S., Zanger P., Campbell O.M.R. (2012). Emergency Obstetric Care Availability: A Critical Assessment of the Current Indicator. Trop. Med. Int. Health.

[25] Banke-Thomas A., Wright K., Sonoiki O., Banke-Thomas O., Ajayi B., Ilozumba O., Akinola O. (2016). Assessing Emergency Obstetric Care Provision in Low- and Middle-Income Countries: A Systematic Review of the Application of Global Guidelines. Glob. Health Action.

[26] Roy L., Biswas T.K., Chowdhury M.E. (2017). Emergency Obstetric and Newborn Care Signal Functions in Public and Private Facilities in Bangladesh. PLoS ONE.

[27] Onambele L., Ortega-Leon W., Guillen-Aguinaga S., Forjaz M.J., Yoseph A., Guillen-Aguinaga L., Alas-Brun R., Arnedo-Pena A., Aguinaga-Ontoso I., Guillen-Grima F. (2022). Maternal Mortality in Africa: Regional Trends (2000–2017). Int. J. Environ. Res. Public Health.

[28] Aliyu L.D., Kadas A.S., Mohammed A., Abdulllahi H.M., Farouk Z., Usman F., Attah R.A., Yusuf M., Magashi M.K., Miko M. (2023). Impediments to Maternal Mortality Reduction in Africa: A Systemic and Socioeconomic Overview. J. Perinat. Med..

[29] United Nations Africa’s Advances in Maternal, Infant Mortality Face Setbacks: WHO. https://news.un.org/en/story/2022/12/1131242.

[30] Nwagbara U.I., Osuala E.C., Chireshe R., Babatunde G.B., Okeke N.O., Opara N., Hlongwana K.W. (2022). Mapping Evidence on Factors Contributing to Maternal and Child Mortality in Sub-Saharan Africa: A Scoping Review Protocol. PLoS ONE.

[31] Kikuchi K., Yasuoka J., Nanishi K., Ahmed A., Nohara Y., Nishikitani M., Yokota F., Mizutani T., Nakashima N. (2018). Postnatal Care Could Be the Key to Improving the Continuum of Care in Maternal and Child Health in Ratanakiri, Cambodia. PLoS ONE.

[32] World Bank World Bank Open Data. https://data.worldbank.org/.

[33] Hyndman R.J., Athanasopoulos G. (2018). Forecasting: Principles and Practice.

[34] Astolfi R., Lorenzoni L., Oderkirk J. (2012). Informing Policy Makers about Future Health Spending: A Comparative Analysis of Forecasting Methods in OECD Countries. Health Policy.

[35] Alegado R.T., Tumibay G.M. (2019). Forecasting Measles Immunization Coverage Using ARIMA Model. J. Comput. Communicat..

[36] Chumachenko D., Meniailov I., Hrimov A., Lopatka V., Moroz O., Tolstoluzka O. (2021). Simulation and Forecasting of the Influenza Epidemic Process Using Seasonal Autoregressive Integrated Moving Average Model. Radioelectron. Comput. Syst..

[37] Lumbreras-Marquez M.I., Fields K.G., Campos-Zamora M., Rodriguez-Bosch M.R., Rodriguez-Sibaja M.J., Copado-Mendoza D.Y., Acevedo-Gallegos S., Farber M.K. (2021). A Forecast of Maternal Deaths with and without Vaccination of Pregnant Women against COVID-19 in Mexico. Int. J. Gynecol. Obstet..

[38] World Bank Health Nutrition and Population Statistics: Population Estimates and Projections. https://databank.worldbank.org/source/population-estimates-and-projections#.

[39] World Health Organization, World Bank, United Nations Population Fund, U.N.C.F. (UNICEF) (2015). Trends in Maternal Mortality: 1990–2015: Estimates from WHO, UNICEF, UNFPA, World Bank Group and the United Nations Population Division.

[40] African Union (2003). Protocol on the Amendments to the Constitutive Act of the African Union.

[41] African Union (2005). Report of the Meeting of Experts from Member States on the Definition of the African Diaspora.

[42] Esmaeilzadeh N., Shakeri M., Esmaeilzadeh M., Rahmanian V. (2020). ARIMA Models Forecasting the SARS-COV-2 in the Islamic Republic of Iran. Asian Pac. J. Trop. Med..

[43] Becketti S. (2013). Introduction to Time Series Using Stata.

[44] Gelper S., Fried R., Croux C. (2009). Robust Forecasting with Exponential and Holt-Winters Smoothing. J. Forecast.

[45] Taylor S.J., Letham B. (2018). Forecasting at Scale. Am. Stat..

[46] Hyndman R., Athanasopoulos G., Bergmeir C., CaceRes G., Chhay L., O’Hara-Wild M., Petropoulos F., Razbash S., Wang E.Y.F. (2023). Forecasting Functions for Time Series and Linear Models; R Package Version 8.21. https://cran.r-project.org/web/packages/forecast/forecast.pdf.

[47] Hyndman R.J., Khandakar Y. (2008). Automatic Time Series Forecasting: The Forecast Package for R. J. Stat. Softw..

[48] WHO (2022). Atlas of African Health Statistics 2022: Health Situation Analysis of the WHO African Region—Country Profiles.

[49] Musarandega R., Machekano R., Munjanja S.P., Pattinson R. (2022). Methods Used to Measure Maternal Mortality in Sub-Saharan Africa from 1980 to 2020: A Systematic Literature Review. Int. J. Gynecol. Obstet..

[50] Arba M.A., Darebo T.D., Koyira M.M. (2016). Institutional Delivery Service Utilization among Women from Rural Districts of Wolaita and Dawro Zones, Southern Ethiopia; a Community Based Cross-Sectional Study. PLoS ONE.

[51] Kolleh E.M., Bestman P.L., Bajinka O., Weamie S.J., Luo J. (2022). Maternal Mortality and Its Risk Factors in Africa: A Systematic Review and Meta-Analysis. Arch. Clin. Obstet. Gynecol. Res..

[52] Ye Z. (2019). Air Pollutants Prediction in Shenzhen Based on ARIMA and Prophet Method. E3S Web Conf..

[53] Geurts M., Box G.E.P., Jenkins G.M. (1977). Time Series Analysis: Forecasting and Control.

[54] Chatfield C. (1996). The Analysis of Time Series.

[55] NIST/SEMATECH e-Handbook of Statistical Methods. https://www.itl.nist.gov/div898/handbook/index.html.

[56] Stellwagen E., Tashman L. (2013). ARIMA: The Models of Box and Jenkins. Foresight: Int. J. Appl. Forecast..

[57] Tsala Dimbuene Z., Amo-Adjei J., Amugsi D., Mumah J., Izugbara C.O., Beguy D. (2018). Women’s education and utilization of maternal health services in africa: A multi-country and socioeconomic status analysis. J. Biosoc. Sci..

[58] Gnimassou T.O.R.T., Filiz E. (2023). Evaluation of Caesarean ExpendituRes. of Households in Benin. Health Soc. Care Commun..

[59] Idowu A., Israel O.K., Akande R.O. (2022). Access, Perceived Quality and Uptake of Antenatal Services in Urban Communities of Osun State, Southwest Nigeria. Afr. J. Reprod. Health.

[60] De Groot A., Van de Munt L., Boateng D., Savitri A.I., Antwi E., Bolten N., Klipstein-Grobusch K., Uiterwaal C.S.P.M., Browne J.L. (2019). Equity in Maternal Health Outcomes in a Middle-Income Urban Setting: A Cohort Study. Reprod. Health.

[61] Adu J., Tenkorang E., Banchani E., Allison J., Mulay S. (2018). The Effects of Individual and Community-Level Factors on Maternal Health Outcomes in Ghana. PLoS ONE.

[62] Hernandez J.C., Moser C.M. (2013). Community Level Risk Factors for Maternal Mortality in Madagascar. Afr. J. Reprod. Health.

[63] Baral Y.R., Lyons K., Skinner J., van Teijlingen E.R. (2012). Maternal Health Services Utilisation in Nepal: Progress in the New Millennium?. Health Sci. J..

[64] Ononokpono D.N., Odimegwu C.O. (2014). Determinants of Maternal Health Care Utilization in Nigeria: A Multilevel Approach. Pan Afr. Med J..

[65] Lan C.-W., Tavrow P. (2017). Composite MeasuRes. of Women’s Empowerment and Their Association with Maternal Mortality in Low-Income Countries. BMC Pregnancy Childbirth.

[66] Alvarez J.L., Gil R., Hernández V., Gil A. (2009). Factors Associated with Maternal Mortality in Sub-Saharan Africa: An Ecological Study. BMC Public Health.

[67] Hamal M., Dieleman M., De Brouwere V., de Cock Buning T. (2020). Social Determinants of Maternal Health: A Scoping Review of Factors Influencing Maternal Mortality and Maternal Health Service Use in India. Public Health Rev.

[68] Cameron L., Contreras Suarez D., Cornwell K. (2019). Understanding the Determinants of Maternal Mortality: An Observational Study Using the Indonesian Population Census. PLoS ONE.

[69] Olonade O., Olawande T.I., Alabi O.J., Imhonopi D. (2019). Maternal Mortality and Maternal Health Care in Nigeria: Implications for Socio-Economic Development. Open Access Maced. J. Med. Sci..

[70] Ganle J.K., Parker M., Fitzpatrick R., Otupiri E. (2014). A Qualitative Study of Health System Barriers to Accessibility and Utilization of Maternal and Newborn Healthcare Services in Ghana after User-Fee Abolition. BMC Pregnancy Childbirth.

[71] Kamal T. (2023). Policy Analysis to Assess the Impact of Inadequate Emergency Obstetric Care (EmOC) Services on Maternal Mortality in Balochistan Pakistan. Populat. Med..

[72] Liang J., Dai L., Zhu J., Li X., Zeng W., Wang H., Li Q., Li M., Zhou R., Wang Y. (2011). Preventable Maternal Mortality: Geographic/Rural-Urban Differences and Associated Factors from the Population-Based Maternal Mortality Surveillance System in China. BMC Public Health.

[73] Mbizvo M.T., Say L. (2012). Global Progress and Potentially Effective Policy Responses to Reduce Maternal Mortality. Int. J. Gynecol. Obstet..

[74] Hanif M., Khalid S., Rasul A., Mahmood K. (2022). Maternal Mortality in Rural Areas of Pakistan: Challenges and Prospects. Rural Health.

[75] Chinn J.J., Eisenberg E., Artis Dickerson S., King R.B., Chakhtoura N., Lim I.A.L., Grantz K.L., Lamar C., Bianchi D.W. (2020). Maternal Mortality in the United States: Research Gaps, Opportunities, and Priorities. Am. J. Obstet. Gynecol..

[76] Anwar J., Torvaldsen S., Morrell S., Taylor R. (2023). Maternal Mortality in a Rural District of Pakistan and Contributing Factors. Matern. Child Health J..

[77] Cham M., Sundby J., Vangen S. (2005). Maternal Mortality in the Rural Gambia, a Qualitative Study on Access to Emergency Obstetric Care. Reprod. Health.

[78] Abouchadi S., Godin I., Zhang W.-H., De Brouwere V. (2022). Eight-Year Experience of Maternal Death Surveillance in Morocco: Qualitative Study of Stakeholders’ Views at a Subnational Level. BMC Public Health.

[79] Metwally A.M., Abdel-Latif G.A., Mohsen A., El Etreby L., Elmosalami D.M., Saleh R.M., El-Sonbaty M.M., Amer H.A., El Deeb S.E., Fathy A.M. (2020). Strengths of Community and Health Facilities Based Interventions in Improving Women and Adolescents’ Care Seeking Behaviors as Approaches for Reducing Maternal Mortality and Improving Birth Outcome among Low Income Communities of Egypt. BMC Health Serv. Res..

[80] Metwally A.M., Saleh R.M., El-Etreby L.A., Salama S.I., Aboulghate A., Amer H.A., Fathy A.M., Yousry R., El-Deeb S.E., Abdel-Latif G.A. (2019). Enhancing the Value of Women’s Reproductive Rights through Community Based Interventions in Upper Egypt Governorates: A Randomized Interventional Study. Int. J. Equity Health.

[81] Limam M., Hachani F., El Ghardallou M., Bachraoui M., Mellouli M., Mtiraoui A., Khairi H., Ajmi T., Zedini C. (2021). Availability, Utilization and Quality of Emergency Obstetric Care Services in Sousse, Tunisia. Pan Afr. Med. J..

[82] Afifi M., El-Adawy M., Hajjeh R. (2022). Women’s Health in the Eastern Mediterranean Region: Time for a Paradigm Shift. East. Medit. Health J..

[83] Bjegovic-Mikanovic V., Abousbie Z.A.S., Breckenkamp J., Wenzel H., Broniatowski R., Nelson C., Vukovic D., Laaser U. (2019). A Gap Analysis of SDG 3 and MDG 4/5mortality Health Targets in the Six Arabic Countries of North Africa: Egypt, Libya, Tunisia, Algeria, Morocco, and Mauritania. Libyan J. Med..

[84] Njah M., Mahjoub M., Atif M.-L., Belouali R. (2018). Maternal Mortality in Maghreb: Problems and Challenges of Public Health. Tunis Med..

[85] Chol C., Negin J., Garcia-Basteiro A., Gebrehiwot T.G., Debru B., Chimpolo M., Agho K., Cumming R.G., Abimbola S. (2018). Health System Reforms in Five Sub-Saharan African Countries That Experienced Major Armed Conflicts (Wars) during 1990–2015: A Literature Review. Glob. Health Act..

[86] Sheikh N.S., Gele A. (2023). Factors Influencing the Motivation of Maternal Health Workers in Conflict Setting of Mogadishu, Somalia. PLoS Glob. Public Health.

[87] Schmeer K.K., Echave P.A., Nyseth Nzitatira H. (2023). Exposure to Armed Conflict and HIV Risk Among Rwandan Women. Demography.

[88] Melberg A., Mirkuzie A.H., Sisay T.A., Sisay M.M., Moland K.M. (2019). ‘Maternal Deaths Should Simply Be 0’: Politicization of Maternal Death Reporting and Review Processes in Ethiopia. Health Policy Plan.

[89] Awoke S.M., Tesfaw L.M., Derebe M.A., Fenta H.M. (2023). Spatiotemporal Distribution and Bivariate Binary Analysis of Antenatal and Delivery Care Utilizations in Ethiopia: EDHS 2000–2016. BMC Public Health.

[90] Han D., Clarke-Deelder E., Miller N., Opondo K., Burke T., Oguttu M., McConnell M., Cohen J. (2023). Health Care Provider Decision-Making and the Quality of Maternity Care: An Analysis of Postpartum Care in Kenyan Hospitals. Soc. Sci. Med..

[91] Ersdal H., Mdoe P., Mduma E., Moshiro R., Guga G., Kvaløy J.T., Bundala F., Marwa B., Kamala B. (2023). “Safer Births Bundle of Care” Implementation and Perinatal Impact at 30 Hospitals in Tanzania—Halfway Evaluation. Children.

[92] Nabulo H., Gottfredsdottir H., Joseph N., Kaye D.K. (2023). Experiences of Referral with an Obstetric Emergency: Voices of Women Admitted at Mbarara Regional Referral Hospital, South Western Uganda. BMC Pregnancy Childbirth.

[93] Poppens M., Oke R., Carvalho M., Ledesma Y., Okullu S., Ariokot M.G., Agwang E., Ekuchu P., Wange H., Boeck M. (2023). In-Hospital Obstetric Delays in Rural Uganda: A Cross-Sectional Analysis of a Hospital Cohort. World J. Surg..

[94] Oluoch-Aridi J., Wafula F., Kokwaro G., Mcalhaney M., Adam M.B. (2021). Understanding Women’s Choices: How Women’s Perceptions of Quality of Care Influences Place of Delivery in a Rural Sub-County in Kenya. A Qualitative Study. Matern. Child Health J..

[95] Alaleit O.D., Kajjimu J., Joseph K., Namirembe M.S., Agaba P.K., Kintu A. (2023). Description and Analysis of the Emergency Obstetric Interfacility Ambulance Transfers (IFTs) to Kawempe National Referral Hospital in Uganda. Afr. J. Emerg. Med..

[96] Berihun A., Abebo T.A., Aseffa B.M., Simachew Y., Jisso M., Shiferaw Y. (2023). Third Delay and Associated Factors among Women Who Gave Birth at Public Health Facilities of Gurage Zone, Southern Ethiopia. BMC Women’s Health.

[97] Masaba B.B., Mmusi-Phetoe R. (2023). A Strategy for Reducing Maternal Mortality in Rural Kenya. Int. J. Women’s Health.

[98] Shimpuku Y., Mwilike B., Mwakawanga D., Ito K., Hirose N., Kubota K. (2023). Development and Pilot Test of a Smartphone App for Midwifery Care in Tanzania: A Comparative Cross-Sectional Study. PLoS ONE.

[99] McLaughlin E., Nagy M., Magorwa J.-B., Kibinakanwa G., McLaughlin R. (2023). Decreasing Urgent Repeat Cesarean Sections by Offering Complimentary Ultrasounds and Consultation in Rural Burundi: The Zigama Mama Project. Front. Glob. Women’s Health.

[100] Ouédraogo O.M.A.A., Ouédraogo C.M.R., Kouanda S. (2022). Discontinuation of the Maternal Death Surveillance and Response System in the Post-conflict Context of the Central African Republic. Int. J. Gynecol. Obstet..

[101] Congo B., Yaméogo W.M.E., Millogo T., Compaoré R., Tougri H., Ouédraogo C.M.R., Kouanda S. (2022). Barriers to the Implementation of Quality Maternal Death Reviews in Health Districts in Burkina Faso. Int. J. Gynecol. Obstet..

[102] Ntambue A.M., Malonga F.K., Cowgill K.D., Dramaix-Wilmet M., Donnen P. (2017). Emergency Obstetric and Neonatal Care Availability, Use, and Quality: A Cross-Sectional Study in the City of Lubumbashi, Democratic Republic of the Congo, 2011. BMC Pregnancy Childbirth.

[103] Ameyaw E.K., Dickson K.S., Adde K.S., Ezezika O. (2021). Do Women Empowerment Indicators Predict Receipt of Quality Antenatal Care in Cameroon? Evidence from a Nationwide Survey. BMC Women’s Health.

[104] Nyabadza F., Mukandavire Z., Hove-Musekwa S.D. (2011). Modelling the HIV/AIDS Epidemic Trends in South Africa: Insights from a Simple Mathematical Model. Nonlinear Anal. Real World Appl..

[105] Marks S. (2002). An Epidemic Waiting to Happen? The Spread of HIV/AIDS in South Africa in Social and Historical Perspective. Afr. Stud..

[106] Adegoke Y.O., Mbonigaba J., George G. (2022). Macro-Economic Determinants, Maternal and Infant SDG Targets in Nigeria: Correlation and Predictive Modeling. Front. Public Health.

[107] Adu J., Owusu M.F. (2023). How Do We Improve Maternal and Child Health Outcomes in Ghana?. Int. J. Health Plan. Manag..

[108] Bershteyn A., Resar D., Kim H.-Y., Platais I., Mullick S. (2023). Optimizing the Pipeline of Multipurpose Prevention Technologies: Opportunities across Women’s Reproductive Lifespans. Front. Reprod. Health.

[109] Tegegne K.T., Wudu T.K., Abdisa B.G., Tegegne E.T., Tessema M.K. (2023). Institutional Delivery Knowledge, Attitude, and Practice among Mothers of Childbearing Age with One or More Children, Ethiopia. J. Prev. Med. Hyg..

[110] Siphiwe Bridget Pearl Thwala (2020). Health Systems Factors Influencing the Delivery of Emergency Obstetric Care in a South African District.

[111] Thwala S.B.P., Blaauw D., Ssengooba F. (2018). Measuring the Preparedness of Health Facilities to Deliver Emergency Obstetric Care in a South African District. PLoS ONE.

[112] Jejaw M., Debie A., Yazachew L., Teshale G. (2023). Comprehensive Emergency Management of Obstetric and Newborn Care Program Implementation at University of Gondar Comprehensive Specialized Hospital, Northwest Ethiopia, 2021: An Evaluation Study. Reprod. Health.

[113] Gazeley U., Reniers G., Romero-Prieto J.E., Calvert C., Jasseh M., Herbst K., Khagayi S., Obor D., Kwaro D., Dube A. (2023). Pregnancy-related Mortality up to 1 Year Postpartum in Sub-Saharan Africa: An Analysis of Verbal Autopsy Data from Six Countries. BJOG.

[114] Zelka M.A., Yalew A.W., Debelew G.T. (2023). Effectiveness of a Continuum of Care in Maternal Health Services on the Reduction of Maternal and Neonatal Mortality: Systematic Review and Meta-Analysis. Heliyon.

[115] Africa Leadership Meeting: Investing in Health | African Union. https://au.int/en/newsevents/20190209/africa-leadership-meeting-investing-health.

[116] WHO (2022). Global Spending on Health: Rising to the Pandemic’s Challenges.

[117] WHO Global Health Expenditure Database. https://apps.who.int/nha/database/.

[118] Alabi Q.K., Oyedeji A.S., Kayode O.O., Kajewole-Alabi D.I. (2023). Impact of COVID-19 Pandemic on Mother and Child Health in Sub-Saharan Africa—A Review. Pediatr. Res..

[119] Kotlar B., Gerson E., Petrillo S., Langer A., Tiemeier H. (2021). The Impact of the COVID-19 Pandemic on Maternal and Perinatal Health: A Scoping Review. Reprod. Health.

[120] Roberton T., Carter E.D., Chou V.B., Stegmuller A.R., Jackson B.D., Tam Y., Sawadogo-Lewis T., Walker N. (2020). Early Estimates of the Indirect Effects of the COVID-19 Pandemic on Maternal and Child Mortality in Low-Income and Middle-Income Countries: A Modelling Study. Lancet Glob. Health.

[121] Banke-Thomas A., Semaan A., Amongin D., Babah O., Dioubate N., Kikula A., Nakubulwa S., Ogein O., Adroma M., Anzo Adiga W. (2022). A Mixed-Methods Study of Maternal Health Care Utilisation in Six Referral Hospitals in Four Sub-Saharan African Countries before and during the COVID-19 Pandemic. BMJ Glob Health.

